# Hydroxyapatite-filled osteoinductive and piezoelectric nanofibers for bone tissue engineering

**DOI:** 10.1080/14686996.2023.2242242

**Published:** 2023-08-24

**Authors:** Frederico Barbosa, Fábio F. F. Garrudo, Paola S. Alberte, Leonor Resina, Marta S. Carvalho, Akhil Jain, Ana C. Marques, Francesc Estrany, Frankie J. Rawson, Carlos Aléman, Frederico Castelo Ferreira, João C. Silva

**Affiliations:** a Department of Bioengineering and iBB-Institute for Bioengineering and Biosciences, Instituto Superior Técnico, Universidade de Lisboa, Lisboa, Portugal; b Associate Laboratory i4HB – Institute for Health and Bioeconomy, Instituto Superior Técnico, Universidade de Lisboa, Lisboa, Portugal; c Department of Bioengineering and Instituto de Telecomunicações, Instituto Superior Técnico, Universidade de Lisboa, Lisboa, Portugal; d Departament d’Enginyeria Química and Barcelona Research Center for Multiscale Science and Engineering, EEBE, Universitat Politècnica de Catalunya, Barcelona, Spain; e Bioelectronics Laboratory, Regenerative Medicine and Cellular Therapies, School of Pharmacy, Biodiscovery Institute, University of Nottingham, Nottingham, UK; f CERENA, Department of Chemical Engineering, Instituto Superior Técnico, Universidade de Lisboa, Lisboa, Portugal; g Institute for Bioengineering of Catalonia (IBEC), The Barcelona Institute of Science and Technology, Barcelona, Spain

**Keywords:** Bone tissue engineering, piezoelectricity, electrospinning, PVDF-TrFE, hydroxyapatite

## Abstract

Osteoporotic-related fractures are among the leading causes of chronic disease morbidity in Europe and in the US. While a significant percentage of fractures can be repaired naturally, in delayed-union and non-union fractures surgical intervention is necessary for proper bone regeneration. Given the current lack of optimized clinical techniques to adequately address this issue, bone tissue engineering (BTE) strategies focusing on the development of scaffolds for temporarily replacing damaged bone and supporting its regeneration process have been gaining interest. The piezoelectric properties of bone, which have an important role in tissue homeostasis and regeneration, have been frequently neglected in the design of BTE scaffolds. Therefore, in this study, we developed novel hydroxyapatite (HAp)-filled osteoinductive and piezoelectric poly(vinylidene fluoride-co-tetrafluoroethylene) (PVDF-TrFE) nanofibers via electrospinning capable of replicating the tissue’s fibrous extracellular matrix (ECM) composition and native piezoelectric properties. The developed PVDF-TrFE/HAp nanofibers had biomimetic collagen fibril-like diameters, as well as enhanced piezoelectric and surface properties, which translated into a better capacity to assist the mineralization process and cell proliferation. The biological cues provided by the HAp nanoparticles enhanced the osteogenic differentiation of seeded human mesenchymal stem/stromal cells (MSCs) as observed by the increased ALP activity, cell-secreted calcium deposition and osteogenic gene expression levels observed for the HAp-containing fibers. Overall, our findings describe the potential of combining PVDF-TrFE and HAp for developing electroactive and osteoinductive nanofibers capable of supporting bone tissue regeneration.

## IMPACT STATEMENT

In this study entitled “***Hydroxyapatite-filled osteoinductive and piezoelectric nanofibers for bone tissue engineering***”, we describe the development of novel hydroxyapatite (HAp)-filled osteoinductive piezoelectric poly(vinylidene fluoride-cotetrafluoroethylene) (PVDF-TrFE) electrospun nanofibers as a potential strategy for supporting bone repair in delayed-union and non-union osteoporotic-related fractures, for which current clinical techniques have proven to be largely inadequate and scaffold-based tissue engineering approaches hold significant promise. While the piezoelectric properties of native bone tissue have been extensively discussed in the literature, including their key role in preserving tissue homeostasis and promoting tissue repair, they have been widely neglected in the design of scaffolds for bone tissue engineering (BTE) applications. Piezoelectric scaffolds can be used not only for mimicking the native piezoelectric features of bone but also to provide a platform for applying electrical or mechanical stimuli to damaged tissue, contributing to an accelerated regeneration process.

The nanofibrous scaffolds generated in this study were capable of replicating the main electrical, structural and compositional properties of bone extracellular matrix (ECM). To the best of our knowledge, this was the first time that the combination of HAp with the piezoelectric polymer PVDF-TrFE was found to induce key shifts in the chemical structure of the polymer and promote β phase nucleation, not only enhancing the piezoelectric features of the constructs but also improving their surface properties, including their ability to support mineralization in vitro. The HAp nanoparticles also provided meaningful bone-like biological cues (osteoinduction), enhancing the osteogenic differentiation of seeded human mesenchymal stem/stromal cells (hMSCs), which was confirmed by an increased ALP activity, cellderived calcium deposition and expression of important osteogenic gene markers. Overall, our findings highlight, for the first time, the potential of combining PVDFTrFE and HAp for developing electroactive and osteoinductive nanofibrous constructs with improved piezoelectric properties, surface features and osteogenic potential capable of improving bone tissue regeneration.

## GRAPHICAL ABSTRACT

**Figure:**
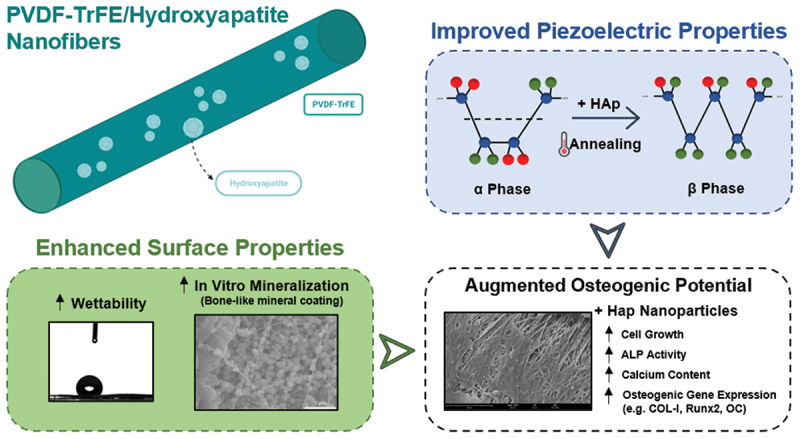

## 1. Introduction

Osteoporosis is a major bone degenerative disorder characterized by the loss of bone mass and the deterioration of the native architectural features of the bone tissue. Osteoporosis is responsible for increasing bone fragility as well as providing a heightened risk of fracture formation [1]. This disorder has a significant prevalence worldwide, being estimated to affect a total of 200 million people as of 2017 and impacting disproportionately the female population [2]. Additionally, osteoporosis has a significant economic impact in developed nations due to the high direct and indirect healthcare expenditures involved with its treatment, such as hospital admissions or pharmacological regimens, and productivity losses, respectively. The annual economic impact with osteoporotic-related fractures is estimated to be €37 billion in the EU alone [1,3].

In most instances, bone fracture repair occurs naturally given the tissue’s innate ability to self-repair. The repair process involves two subsequent anabolic and catabolic phases in which stem cells are recruited to differentiate into skeletal and vasculature tissue. Following this, a temporary cartilaginous callus is generated in the lesion site, which is later mineralized and transformed into early immature bone tissue [4]. However, for delayed union and non-union fractures, which have been reported in approximately 5% to 10% of the cases, the bone is unable to properly regenerate itself. This could be attributed to either a lack of mechanical stability (hypertrophic fracture) or reduced callus formation (atrophic fracture) [5]. For these particular instances, clinical intervention becomes necessary (e.g. autografts, allografts). Given the uneven and suboptimal results obtained with current therapeutic strategies, bone tissue engineering (BTE) approaches involving the fabrication of scaffolds for temporarily replacing damaged bone tissue and promoting its regeneration are gaining interest. BTE strategies present a number of key advantages compared with other techniques (e.g. autografts), including the absence of issues related with reduced graft availability or complications commonly associated with autologous bone harvesting. The produced synthetic scaffolds are also more reproducible (high degree of control over scaffold features) and can be easily customized for patient-specific applications [6,7].

Similar to other connective tissues, such as cartilage, the bone tissue is piezoelectric, i.e. by applying mechanical loads to the native tissue, an electrical signal is generated within the bone’s extracellular matrix (ECM). The piezoelectric The piezoelectric coefficient (d33) of bone tissue (...) has been reported to be between 0.7−2.3pC/N [8,9]. This electrical property of the bone stems primarily from the significant presence of type I collagen fibers in the bone ECM, which are inherently piezoelectric due to the interactions of -CO and -NH polar groups in the protein’s glycine, proline and hydroxyproline residues [8,10]. Hydroxyapatite (HAp), a major component of the inorganic phase of bone ECM (70–90%), has also been found to contribute to the tissue’s piezoelectricity [11,12]. Its piezoelectric properties stem from ion displacements in the crystalline structure as a result of applied mechanical stress, which shift the balance of ions in the structure and lead to the creation of a dipole moment. The presence of symmetry breaks in the centrosymmetric HAp structure (at nanoscale dimensions or nonequilibrium conditions) allows a net polarization to develop and an electrical signal to be generated [11,13]. Due to its direct involvement in bone remodeling and tissue regeneration processes, the bone’s piezoelectric property plays a critical role in bone homeostasis. Mechanical loads placed on the joint and transmitted to the underlying bone tissue cause electrical stimuli to be produced within the bone’s ECM, which leads to the mobilization of osteoblasts and encourages the production of new bone, improving the tissue’s mechanical response to loads [14]. Several studies have also identified the positive contribution of applying exogenous electrical stimuli on promoting bone ECM formation and fracture repair [15–18]. Therefore, the development of piezoelectric scaffolds for BTE is of interest, because such constructs could be used to not only mimic the connective tissue’s inherent piezoelectricity but also to provide a platform for applying direct electrical stimulation to damaged bone tissue. In doing so, bone regeneration can be further enhanced without the need to implant potentially harmful electrodes. However, the creation of such scaffolds in the context of BTE is still substantially unexplored.

A significant array of smart piezoelectric biomaterials has been reported in the literature for developing electroactive scaffolds for TE applications. They can be subdivided into two main categories: piezoceramics and piezoelectric polymers (PZPs) [14,19]. The synthetic PZP polyvinylidene fluoride (PVDF) and its derivatives, namely poly(vinylidene fluoride-co-tetrafluoroethylene) (PVDF-TrFE), have been widely applied in TE as a result of their high piezoelectric coefficient, excellent biocompatibility and physicochemical features, and ease of processing. PVDF-TrFE comprises a non-piezoelectric α and piezoelectric β (more prominent) crystalline phases, characterized by polar fluorine groups with antiparallel and parallel configurations, respectively. The relative balance between these two phases affects the overall piezoelectric properties of the polymer. Many natural biodegradable piezoceramics and PZPs, such as HAp, collagen and chitosan, have also been applied in BTE, even though in most instances their use has been primarily related to their excellent biological features, as their intrinsic piezoelectric properties tend to be neglected for the most part [19].

Electrospinning, a technique used to produce fibers with diameters in the micrometer and nanometer range, has been applied in BTE to develop scaffolds with a high surface-to-volume ratio capable of emulating the fibrous nature of the bone tissue’s ECM [20]. The resulting constructs can mimic relevant nano- and micro-sized features of the bone, potentially improving tissue regeneration by providing more native-like biomechanical cues as well as increasing cell attachment and growth [21]. Recently, Wu *et al.* (2021) developed osteoblast-derived ECM coated poly(l-lactic acid) (PLLA)/silk fibroin (SF) nanofibers, combining the excellent mechanical properties of PLLA with the biocompatibility and hydrophilicity of SF. These fibers displayed an augmented osteogenic potential compared to the non-coated scaffolds or the osteoblast-derived ECM alone [22]. Piezoelectric fibrous scaffolds, using both piezoceramics and PZPs, have also been developed for BTE, albeit to a smaller extent. Nagarajan *et al.* (2017) fabricated boron-nitride-filled gelatin piezoelectric nanofibers that were able to induce the expression of osteogenic markers by osteosarcoma cells in the absence of growth factors in the cell culture medium [23]. Kitsara *et al.* (2019) developed oxygen plasma-treated permanently hydrophilic PVDF fibers using a rotating drum collector, which supported an increased proliferation and integration of human osteosarcoma cells [24]. A comprehensive review of the piezoelectric fibrous scaffolds developed in the context of BTE is provided elsewhere [19].

This work aimed to develop and optimize novel bioactive piezoelectric PVDF-TrFE/HAp nanofibers, generated through electrospinning, to recapitulate some of the main electrical, structural and compositional features of the bone tissue’s ECM. The resulting composite scaffolds were characterized in terms of their structural, piezoelectrical and mechanical properties. Different annealing strategies were trialed to augment the piezoelectricity of the fibrous scaffolds. The electroactive nanofibers were seeded with human mesenchymal stem/stromal cells (MSCs) to assess their ability to support osteogenic differentiation. To the best of our knowledge, this is the first study where PVDF-TrFE and HAp were combined to develop osteoinductive piezoelectric nanofibers for BTE.

## 2. Materials and methods

### 2.1. Materials

PVDF-TrFE (70/30, mol%/mol%) was acquired from Arkema (Colombres, France). Synthetic HAp nanopowder (MW 502.31 Da; <200 nm particle size) was purchased from Sigma-Aldrich. Dimethylformamide (DMF) was obtained from Carlo Erba reagents and acetone (≥99,8%) was acquired from Sigma-Aldrich (St. Louis, Missouri, USA).

### 2.2. Preparation of electrospinning casting solutions

PVDF-TrFE/HAp solutions were prepared by mixing HAp nanoparticles at multiple concentrations − 1%, 3%, 5% and 10% (wt%) – in DMF:Acetone (3:2). The mixture obtained was ultrasonicated (Ultrasonic Cleaner; VWR) and then agitated in a rocking platform (VWR) overnight. The PVDF-TrFE powder was then added at a fixed concentration of 17% (w/v), and the solutions were agitated for a few hours until a final homogeneous dispersion was obtained. Prior to electrospinning, the PVDF-TrFE/HAp solutions were ultrasonicated for 15 min. PVDF-TrFE electrospinning casting solutions (without HAp nanoparticles) were prepared by simply mixing the piezoelectric polymer in DMF:Acetone (3:2), after which the resulting solutions were agitated for a few hours. A summary of the different conditions investigated in this study is presented in Table 1.

**Table 1.** Nomenclature and composition of the solutions used in the preparation of the nanofibers.

Sample	PVDF-TrFE (mg)	HAp (mg)	DMF (mL)	Acetone (mL)
PVDF-TrFE	850	.0	3	2
PVDF-TrFE/HAp 1%	850	8.5	3	2
PVDF-TrFE/HAp 3%	850	25.5	3	2
PVDF-TrFE/HAp 5%	850	42.5	3	2
PVDF-TrFE/HAp 10%	850	85.0	3	2

### 2.3. Electrospinning setup and parameters

An applied voltage of 20 kV and a controlled flow rate of 1 mL/h were used to generate the PVDF-TrFE/HAp nanofibers, creating an electrical potential between a 21 G stainless steel needle and a grounded static copper collector covered with aluminum foil at a distance of 17 cm from the needle tip (SI, Scheme S1). The temperature and relative humidity were monitored and controlled during electrospinning: the parameters varied between 20°C and 22°C and 35% and 55%, respectively.

### 2.4. Fiber post-processing

After being electrospun, the PVDF-TrFE fiber meshes without HAp nanoparticles and with 10% HAp (wt%) were annealed following two distinct procedures (P1 and P2) to increase their β crystalline phase content and, as a result, enhance the piezoelectricity of the fibers. In the first procedure (P1), adapted from Lam *et al.* (2019), the piezoelectric nanofibers were first placed in a mechanical convection oven at 120°C for 2 h, after which the oven was turned off and the fibers were cooled down to room temperature (corresponding to a cooling rate of ca. 2°C min^−1^ decrease) [25]. In the second annealing procedure (P2), adapted from Satthiyaraju *et al.* (2019), the nanofibers were heated to 100°C for 4 h in the oven, after which a similar cooling process was applied [26].

### 2.5. PVDF-TrFE films

PVDF-TrFE films with and without HAp were prepared by mixing PVDF-TrFE powder (17%, w/v) and HAp nanoparticles at different concentrations (1%, 3%, 5%, 10%, wt%) in DMF:Acetone (3:2). After being agitated overnight in a rocking platform, the solutions were ultrasonicated for 5 min, after which they were deposited onto a glass plate and left to evaporate at room temperature.

### 2.6. Characterization of composite PVDF-TrFE/HAp nanofibers

#### 2.6.1. Scanning electron microscopy (SEM) analysis

The structural characterization of piezoelectric nanofibers was performed using a field emission gun SEM (FEG-SEM) (Model JSM-7001F; JEOL, Tokyo, Japan). Prior to imaging, the samples were mounted on a holder using carbon tape and were coated with a 30 nm gold/palladium (60:40) layer (Model E5100 Sputter Coater; Polaron: Quorum Technologies, Lewes, UK). Using an average accelerating voltage of 15 kV, the samples were imaged at several magnifications. The average fiber diameter of the different non-woven fibrous mats was computed using the ImageJ software (developed by the National Institutes of Health, NIH, U.S.A.): the average diameter of 100 individual nanofibers (n=100) per condition was estimated from at least five different SEM images.

#### 2.6.2. Elemental composition analysis

Energy dispersive X-ray (EDX) analysis (INCA Microanalysis Suite software) of the generated piezoelectric nanofibers was used in order to identify the different elements present in the fibers, therefore corroborating the presence of both PVDF-TrFE and HAp on their composition. EDX was also performed to evaluate whether the *in vitro* mineralization of the composite fibers when exposed to Simulated Body Fluid (SBF) had successfully occurred and to assess the cell-derived mineralization after 3 weeks of MSC osteogenic differentiation on the produced fibrous scaffolds. This analysis was performed with an average acceleration voltage of 10 kV and a spot size of 30 μm.

#### 2.6.3. Atomic force microscopy (AFM) analysis

The roughness of 2 × 2 μm areas of the electrospun fibers was determined by AFM using a Dimension FatScan AFM system (Bruker Corporation, Billerica, Massachusetts, USA) in peak force mode mapping in air at room temperature. SPARK 70 Pt probes (Nunano, Bristol, UK) were used, with a tip radius of curvature <30 nm, a spring constant of 2 N.m^−1^ and a resonant frequency of 70 kHz. Offset images of the fibers’ surface in areas of 0.2 × 0.2 μm were also acquired. Data analysis of the AFM images was performed using the Gwyddion 2.6 software (developed by the Czech Metrology Institute).

#### 2.6.4. Contact angle

The contact angle of the piezoelectric nanofibers was measured by a DSA25B goniometer (Kruss, Hamburg, Germany) using the sessile drop method: distilled water was used for producing the droplets. Droplet spreading on the surface of the different fibrous mats was evaluated by measuring the contact angle of the droplets with the surface. The right and left contact angles were determined, and the average value was computed. A 2 s^−1^ rate of acquisition was used. For each experimental group, the contact angle was imaged and measured in eight independent fiber samples (n=8).

#### 2.6.5. X-ray diffraction (XRD) analysis

XRD analysis was used to corroborate the presence of both PVDF-TrFE and HAp in the composite piezoelectric fibers. The crystalline structure of the samples was analyzed by XRD using a Bruker D8 Advance with Da Vinci diffractometer, with a Cu X-ray tube at 40 kV and 40 mA. The samples were scanned over 2θ range of 10–70° with a step size of 0.02° and a step time of 3 s.

#### 2.6.6. Attenuated total reflection Fourier-transform infrared (ATR-FTIR) spectroscopy

The ATR-FTIR analysis was performed using a Spectrum Two FT-IR Spectrometer from PerkinElmer (Waltham, Massachusetts, U.S.A.), equipped with an attenuated total reflection (ATR) UATR Two accessory. ATR-FTIR spectra were collected to identify important functional groups commonly found in both PVDF-TrFE and HAp to further confirm the presence of these biomaterials within the fiber composition, as well as assess the impact of annealing and the effect of increasing HAp nanoparticle concentration on the β-phase content of the resulting PVDF-TrFE nanofibers. Transmittance spectra were obtained at room temperature over the spectral region from 400 cm^−1^ to 4000 cm^−1^, with a resolution of 4 cm^−1^ and eight scans of data accumulation. All ATR-FTIR spectra were normalized using the maximum and minimum transmittance of each spectrum.

#### 2.6.7. Relative β-phase content estimation

The relative β-phase fractions of the as-spun and annealed PVDF-TrFE nanofibers with and without HAp were estimated by a quantitative comparison of the absorbance values registered at 765 cm^−1^ and 850 cm^−1^ in the ATR-FTIR spectra, which are attributed to the non-polar α-phase and the polar β-phase of PVDF-TrFE, respectively. The following equation, first described by Gregorio *et al.* (1994) and based on the Beer–Lambert Law, was used [27]:

(1) $$F\left(\beta \right)=\frac{A_{\beta }}{\left(\frac{K_{\beta }}{K_{\alpha }}\right)A_{\alpha }+A_{\beta }}$$

Where F(β) corresponds to the relative β-phase fraction of PVDF-TrFE, $A_{\beta }$ and $A_{\alpha }$ correspond to the absorbance values registered in the ATR-FTIR spectra at the β (850 cm^−1^) and α (765 cm^−1^) peaks of PVDF-TrFE, and $K_{\beta }$ and $K_{\alpha }$ are the absorption coefficients at the respective wavenumbers, with values given as $7.7\times 10^{4}\;$cm^2^/mol for 850 cm^−1^ and $6.1\times 10^{4}$ cm^2^/mol for 765 cm^−1^ [28].

Moreover, the collected XRD data was also used to estimate the relative β-phase content of the fabricated scaffolds. The following equation was applied [29]:

(2) $$F\left(\beta \right)=\frac{I_{\left(200/110\right)}+I_{\left(101\right)}+I_{\left(221\right)}}{I_{\left(110\right)}+I_{\left(002\right)}+I_{\left(200/110\right)}+I_{\left(101\right)}+I_{\left(221\right)}}$$

Where F(β) corresponds to the relative β-phase fraction of PVDF-TrFE, $I_{\left(200/110\right)}$, $I_{\left(101\right)}$ and $I_{\left(221\right)}$ correspond to the diffraction peak intensities measured at 2Θ values of 20.6°, 36.2° and 56°, respectively (related with the (200)/(110), (101) and (221) lattice planes of the β polymorph), $I_{\left(110\right)}$ corresponds to the diffraction peak intensity measured at 2Θ value of 20.2° (related with the (110) lattice plane of the γ polymorph), and $I_{\left(002\right)}$ corresponds to the diffraction peak intensity measured at 2Θ value of 39° (related with the (110) lattice plane of the α polymorph) [29].

#### 2.6.8. Quasi-static piezoelectric charge coefficient (d_33_) measurement

The piezoelectric charge coefficient (d_33_) of the as-spun PVDF-TrFE nanofibers with and without HAp was measured using a PKD3–2000-F10N quasi-static piezoelectric meter (PolyK, State College, PA, U.S.A.) according to the Berlincourt method. Three independent 1.5 × 1.5 cm^2^ samples of each experimental condition were used. Measurements were performed at a calibrated force of 0.30 N (110 Hz). The temperature and relative humidity were monitored during the piezoelectric charge coefficient measurements: the parameters remained mostly stable at 25°C and 52%, respectively. A photo of the setup used can be found in Figure 3(a).

#### 2.6.9. Piezoresistivity assay

Electroconductivity measurements of 0.5 × 3 cm^2^ films were performed using a digital multimeter (Mod.60.101, Electro DH, Spain) connected to the sample by two clips during deformation (see Figure 3(c,d)). The deformation was performed at room temperature and humidity using a universal tensile testing instrument Zwick Z2.5/TN1S with a 100 N load cell equipped with a testXpert 8.1 program as in previous studies [30]. Initial distance between clamps was 10 mm and the crosshead speed was set to 10 mm/min.

#### 2.6.10. Mechanical properties

The mechanical properties of the as-spun and annealed PVDF-TrFE and PVDF-TrFE/HAp electrospun scaffolds were tested under uniaxial tensile testing at room temperature using a mechanical tester (Model UV-200-01; CellScale Biomaterials Testing, Waterloo, Canada) with a 10 N load cell and a displacement rate of 3 mm/min. Prior to testing, 10 specimens from each experimental group (n=10) were cut into rectangular strips and their thickness, width and length were measured. Experimental data was collected and processed using the UniVert software. Stress–strain curves were obtained by considering samples’ cross-section areas and initial lengths. After plotting the stress–strain curves, the Young’s modulus (MPa) of the fibers was computed from the 0–15% initial linear region slope, while the ultimate tensile strength (UTS) (MPa) of each sample was obtained from the highest peak of each curve. Ultimate elongation (%) was determined by dividing the displacement measured at the highest peak of the stress–strain curve by the original length of each specimen.

### 2.7. In vitro fiber mineralization

*In vitro* mineralization of the nanofibrous scaffolds was conducted to assess their ability to promote the formation of a biologically appropriate mineral coating and, therefore, predict the fiber’s *in vivo* bone bioactivity, which constitutes a meaningful property for BTE-related applications [31]. The scaffolds were incubated for a 28-day period in Simulated Body Fluid (SBF), a saline solution with almost identical ionic composition to the blood plasma. SBF solution was prepared according to Kokubo *et al.* (2006) [31]. The fibers were first washed in distilled water and 20% ethanol to remove impurities, after which the scaffolds were immersed in SBF and finally placed in an incubator at 37°C. The medium was changed every 2 days. Mineralized fibers were sampled after 7, 14 and 28 days of incubation to monitor the SBF-mediated mineralization process. After being incubated, the piezoelectric nanofibers were washed with distilled water to remove any soluble salts (e.g. NaCl).

### 2.8. In vitro cell culture studies

#### 2.8.1. Cell culture

The human bone marrow mesenchymal stem cells (hBM-MSCs) used were part of the cell bank available at the Stem Cell Engineering Research Group, Institute for Bioengineering and Biosciences (iBB) at Instituto Superior Técnico (IST). hBM-MSCs were previously isolated according to protocols previously established at iBB-IST [32]. Bone marrow aspirates (Male 46 years) were obtained from Centro Clínico da GNR, Lisboa, under collaboration agreements with iBB-IST. An additional sample of fresh unprocessed bone marrow (Male 24 years) was obtained from Lonza (Switzerland). All human samples were obtained from healthy donors after written informed consent according to Directive 2004/23/EC of the European Parliament and of the Council of 31 March 2004, on setting standards of quality and safety for the donation, procurement, testing, processing, preservation, storage, and distribution of human tissues and cells (Portuguese Law 22/2007, June 29), with the approval of the Ethics Committee of the respective clinical institution. Isolated cells were kept frozen in liquid/vapor nitrogen tanks until further use. Prior to the cell culture assays, the MSCs were thawed and expanded on tissue culture flasks (*T*-75 cm^2^) using low-glucose Dulbecco’s Modified Eagle’s Medium (Gibco DMEM; Thermo Fisher Scientific) supplemented with 10% fetal bovine serum (Gibco FBS; Thermo Fisher Scientific) and 1% antibiotic-antimycotic (Gibco Anti-Anti Solution; Thermo Fisher Scientific). The cells were kept in an incubator at 37°C and 5% CO_2_ in a humidified atmosphere and the medium was exchanged every 3–4 days. All the experiments were conducted using cells between passages 4 and 6.

#### 2.8.2. hBM-MSC seeding and culture on electrospun piezoelectric nanofibers

Prior to cell seeding, the as-spun PVDF-TrFE and PVDF-TrFE/HAp electrospun fibers were collected from the aluminum foil and fixed on glass coverslips (13 mm of diameter; VWR) with FDA-approved biocompatible adhesive silicone glue (Silastic Medical Adhesive Silicone, Type A; Dow Corning). The fibers were left for approximately 24 h to glue properly. Afterwards, the fibers were sterilized by UV light exposure overnight, after which they were placed in ultra-low cell attachment 24-well plates (Corning). The scaffolds were then washed three times with a 1% anti-anti solution, prepared in a phosphate buffer solution (PBS) and incubated in culture medium for 1 hour at 37°C.

After being harvested from the T-flasks, the hBM-MSCs were seeded in the PVDF-TrFE and PVDF-TrFE/HAp fibers at a density of 60.000 cells per scaffold. The cell-seeded scaffolds were incubated at 37°C and 5% CO_2_ in a humidified atmosphere for 2 h without cell culture medium to promote initial cell adhesion. Osteogenic medium composed of DMEM supplemented with 10% FBS (MSC qualified), 10 mM of β-glycerolphosphate (Sigma-Aldrich), 10 nM of dexamethasone (Sigma-Aldrich), 50 μg/mL of ascorbic acid (Sigma-Aldrich) and 1% anti-anti was added to the cell-seeded piezoelectric nanofibers. The culture was conducted during a 21-day period at 37°C and 5% CO_2_ in a humidified atmosphere. Cell media renewal was performed every 3–4 days.

#### 2.8.3. Evaluation of the biological performance and osteogenic potential of the piezoelectric nanofibers

##### 2.8.3.1. hBM-MSC proliferation assay

The proliferation of hBM-MSCs on the piezoelectric fibrous scaffolds was evaluated using the AlamarBlue assay (AlamarBlue Cell Viability Reagent; Thermo Fisher Scientific) following the manufacturer’s guidelines. This non-destructive assay was applied to monitor the metabolic activity of the cells seeded on the piezoelectric scaffolds and respective controls on days 3, 7, 14 and 21 of culture. Briefly, a 10% AlamarBlue solution diluted in cell culture media was added to the scaffolds and incubated at 37°C and 5% CO_2_ in a humidified atmosphere for 3 h. Fluorescence intensity was measured in a microplate reader (Infinite 200 Pro; Tecan, Switzerland) at an excitation/emission wavelength of 560/590 nm. For each experimental group, the fluorescence intensity was analyzed for five independent piezoelectric scaffolds (n=5) and acellular electrospun scaffolds were used as blank controls. Fluorescence intensity values were measured in triplicates for each sample. The obtained fluorescence intensity values were correlated with the number of viable hBM-MSCs present in the different piezoelectric scaffolds through a calibration curve.

##### 2.8.3.2. Cell morphology assessment

In order to assess the morphology, adhesion and distribution of the hBM-MSCs, the samples were stained with 4,6-diamidino-2-phenylindole dihydrochloride (DAPI) and Phalloidin after 21 days of cell culture. First, the cells were washed with PBS and fixed with a 4% paraformaldehyde (PFA, Sigma-Aldrich) solution for 20 min. Then, the cells were permeabilized in 0.1% Triton X-100 (Sigma-Aldrich) for 10 min. After permeabilization, the cells were incubated in the dark with Phalloidin-TRITC (2 μg/mL in PBS) (Sigma-Aldrich) for 45 min. Afterwards, the samples were washed twice with PBS and were counterstained with DAPI (1.5 μg/mL in PBS) (Sigma-Aldrich) for 5 min. The cells were then washed with PBS, and the fluorescence staining was imaged using an inverted fluorescence microscope (LEICA DMI3000B, Leica Microsystems, Wetzlar, Germany).

The morphology of the cells on the piezoelectric electrospun fibers was also observed through SEM imaging after 21 days of cell culture. Previously fixed samples in 4% PFA were dehydrated by immersing the scaffolds in ethanol solutions with increasing concentrations (20%, 40%, 60%, 80%, 90% and 100% (v/v)) for 30 min each. The fibers were then immersed in hexamethyldisilazane (HMDS) for 1 h, after which they were left to air-dry overnight in a fume hood. Prior to SEM imaging, the dried cell-seeded scaffolds were sputter-coated with a gold/palladium (Au/Pd) layer, as previously described.

##### 2.8.3.3. Alkaline phosphatase quantification assay

Alkaline phosphatase (ALP) activity, commonly associated with osteoblast function and bone formation, was quantified using a colorimetric ALP quantification kit (BioAssays Systems), following the manufacturer’s guidelines. ALP activity was assessed for hBM-MSC cultures after 14 and 21 days of osteogenic differentiation on the different electrospun scaffolds. The samples were washed with PBS and incubated in a 0.2% Triton X-100 solution overnight at room temperature. Afterwards, a *p*-nitrophenyl solution (10 mM) was added to the lysates. The absorbance was measured on a microplate reader (Infinite 200 Pro; Tecan) at 405 nm. The values were normalized to the number of MSCs present on each scaffold. For each experimental group, the absorbance was quantified for three independent scaffolds (n=3) and was measured in duplicates for each sample.

##### 2.8.3.4. Calcium content assay

The levels of calcium present in the piezoelectric electrospun scaffolds (due to cell-derived mineralization) after 21 days of osteogenic differentiation were assessed using a calcium colorimetric assay kit (Sigma-Aldrich). First, the samples were washed with PBS and incubated in a 1 M HCl solution overnight (with agitation). Afterwards, the supernatant was collected and used for calcium determination, following the manufacturer’s instructions. Briefly, diluted forms of a Calcium Standard Solution (500 mM) available in the kit were pipetted into a well plate at several concentrations. Three samples of each experimental condition (n=3) were also pipetted into the well plate. Afterwards, a chromogenic reagent and a calcium assay buffer (present in the kit) were added to each well, and the solutions were gently mixed. The samples were incubated in the dark for 5–10 min at room temperature. The absorbance was measured on a microplate reader (Infinite 200 Pro; Tecan) at 575 nm (duplicate measurements per sample). The absorbance measurements for the different calcium standard solution concentrations were used to develop a calibration curve which, in turn, was used to estimate the concentration of calcium present in each sample. The values were normalized to the number of MSCs present on each scaffold. The supernatants of acellular electrospun PVDF-TrFE/HAp scaffolds with varying HAp concentrations were used in this assay as blank controls to eliminate the contribution of calcium from the HAp nanoparticles present in the initial fiber formulation.

##### 2.8.3.5. RNA isolation and quantitative real-time PCR (qRT-PCR) analysis

RNA extraction was performed using the RNeasy Mini Kit (QIAGEN). Briefly, 600 μL of lysis buffer (RLT buffer) was added to each scaffold sample, which were agitated for 1 h. Afterwards, total RNA was isolated and purified following the manufacturer’s guidelines and the RNA concentration was quantified using a NanoVue Plus spectrophotometer (GE Healthcare). After asserting the proper total RNA concentration of each experimental condition (using RNase-free water), cDNA was synthesized from the purified RNA using the High-Capacity cDNA Reverse Transcription Kit (Applied Biosystems) according to the manufacturer’s protocol. Essentially, reaction mixtures comprising 10 μL of MasterMix – constituted by 2 μL of RT 10× buffer, 0.8 μL of dNTP mix, 4.2 μL of RNase-free water, 2 μL of random primers and 1 μL of Multiscribe Reverse Transcriptase – and 10 μL of purified RNA sample were incubated in a T100^TM^ thermal cycler (Bio-Rad). The qRT-PCR analysis was performed using a StepOnePlus real-time PCR system (Applied Biosystems). Reaction mixtures comprising 1 μL (10 ng) of cDNA sample and 19 μL of a MasterMix solution – constituted by 10 μL of ROX plus (Nzytech), 1 μL of FW primer, 1 μL of REV primer and 7 μL of RNase-free water – were prepared for each target gene and experimental condition. All reactions were carried out at 95°C for 10 min, followed by 40 cycles of 95°C for 15 s and 60°C for 1 min. All samples were analyzed in triplicates (n=3). The results obtained were analyzed using the 2^−∆∆Ct^ method to determine relative changes in specific osteogenic marker genes expression compared with the control sample (hBM-MSCs at day 0 before scaffold seeding). Gene expression was primarily normalized to the housekeeping gene glyceraldehyde-3-phosphate (*GADPH*) and then determined as a fold-change relative to the baseline expression of the target genes in the control sample. The primer sequences used in the qRT-PCR analysis are presented in Table 2.

**Table 2.** Primer sequences used in the RT-qPCR analysis.

Gene	Forward primer sequence	Reverse primer sequence
GAPDH	5’-GGTCACCAGGGCTGCTTTTA-3’	5’-CCTGGAAGATGGTGATGGGA-3’
*ALP*	5’-ACCATTCCCACGTCTTCACATTT-3’	5’-AGACATTCTCTCGTTCACCGCC-3’
*Runx2*	5’-AGATGATGACACTGCCACCTCTG-3’	5’-GGGATGAAATGCTTGGGAACT-3’
*COL I*	5’-CATCTCCCCTTCGTTTTTGA-3’	5’-CCAAATCCGATGTTTCTGCT-3’
*OC*	5’-TGTGAGCTCAATCCGGCATGT-3’	5’-CCGATAGGCCTCCTGAAGC-3’
*OPN*	5’-CAGGTCTGCGAAACTTCTTAG-3’	5’-CTCCATTGACTCGAACGACTC-3’
*CACNA1C*	5’-GTACAAAGACGGGGAGGTTGAC-3’	5’-GTAGTTGTAGATGGGGCCCTTG-3’
*CACNA1H*	5’-TCAACGTCATCACCAGTCC-3’	5’-AGCCTCGAAGACAAACACGA-3’

##### 2.8.3.6. Osteogenic staining

ALP/Von Kossa staining, generally performed to confirm osteogenic differentiation (through the detection of early bone progenitors) and identify calcium aggregate formation, was used in this study to assess the osteogenic potential of the PVDF-TrFE-based fibers after 21 days of cell culture. After being washed with PBS and fixed with 2% PFA for 15 min, the hBM-MSCs were first washed twice with Milli-Q, after which the piezoelectric scaffolds were stained for ALP by incubation in a solution comprised by 0.1 M TRIS-HCl (Sigma-Aldrich), containing Fast Violet Solution (Sigma-Aldrich) and Naphthol AS MX-P04 (Sigma-Aldrich) for 45 min. The cells were then washed three times with PBS and kept in Milli-Q water while being observed under a microscope (LEICA DMI3000B, Leica Microsystems). After washing the samples with PBS, Von Kossa staining was performed on the same fibrous scaffolds by incubating them in a 2.5% silver nitrate solution (Sigma-Aldrich) for 30 min. The scaffolds were washed three times with PBS, once with Milli-Q water, and kept in Milli-Q water until observation under the microscope.

Alizarin red staining of the fibrous scaffolds was performed to detect the calcium deposits formed on the fibers as a result of cell-derived mineralization processes. PFA-fixed samples were incubated in a 2% Alizarin red (Sigma-Aldrich) solution (in PBS) for 1 h at room temperature. Afterwards, the scaffolds were washed multiple times with PBS and Milli-Q water, after which, they were imaged with an inverted fluorescence microscope (LEICA DMI3000B, Leica Microsystems).

To further corroborate the presence of mineral deposits within the samples after 21 days of cell culture, formed as a result of the osteogenic differentiation of hBM-MSCs, a 20 mM Xylenol orange solution (Sigma-Aldrich) was added to previously fixed cell-seeded piezoelectric fibers and incubated for 1 h at room temperature. The scaffolds were then washed successively with PBS, counterstained with DAPI (1.5 μg/mL in PBS) for 5 min and washed once again with PBS. The fluorescence staining was imaged using an inverted fluorescence microscope (LEICA DMI3000B, Leica Microsystems).

##### 2.8.3.7. Immunofluorescence analysis

The distribution of type I collagen and osteopontin (important macromolecules produced during bone ECM formation) within the structure of the different piezoelectric scaffolds after 21 days of osteogenic differentiation was evaluated through immunofluorescence analysis. Previously fixed cell-seeded electrospun fibers were washed twice in PBS, after which the scaffolds were immersed in a permeabilization/blocking solution comprising 1% BSA (Sigma-Aldrich), 10% FBS and 0.03% Triton X-100 for 45 min at room temperature. Solutions containing primary antibodies for type I collagen (MA1–26771, Thermo-Fischer) and osteopontin (ab8448, Abcam) (1:200 in 1% BSA, 10% FBS, 0.03% Triton X-100 solution) were then incubated with the respective samples overnight at 4°C. The fibrous scaffolds were then incubated with a secondary antibody (1:200 in 1% BSA) for 1 h at room temperature in the dark. Following two washes with PBS, the fibers were counterstained with DAPI (1.5 μg/mL in PBS) for 5 min at room temperature, washed twice with PBS and imaged using a fluorescence microscope (LEICA DMI3000B, Leica Microsystems).

### 2.9. Statistical analysis

Results are presented as mean values ± standard deviation (SD). All the characterization experiments were conducted using three independent samples (n=3), unless specified otherwise. In vitro cell culture results were obtained from three independent samples (n=3) from two different donors. Statistical analysis of the data was performed by one-way ANOVA, followed by Tukey post-hoc test by the GraphPad Prism 5 software. Data was considered statistically significant when the p-values obtained were less than 0.05 (95% confidence intervals, *p < 0.05).

## 3. Results

### 3.1. Morphological and compositional features of PVDF-TrFE/HAp nanofibers

**Figure 1.**
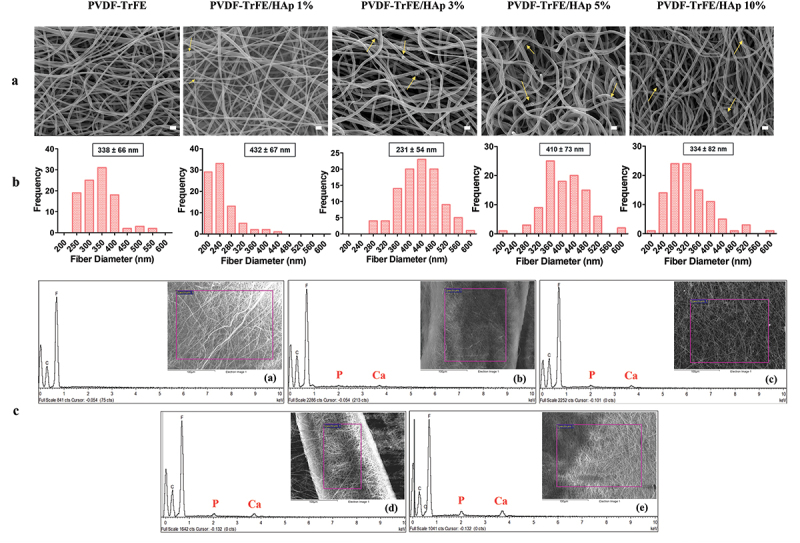
SEM micrographs (a) and respective fiber diameter distribution histograms (b) of the different electrospun PVDF-TrFE and PVDF-TrFE/HAp nanofibers. The yellow arrows identify HAp aggregates present in each fiber condition. Scale bar: 1 μm. Elemental composition analysis (c) of the generated composite scaffolds: PVDF-TrFE fibers without HAp (a) and PVDF-TrFE fibers functionalized with 1% (b), 3% (c), 5% (d) and 10% (e) HAp. EDX spectra were collected on non-coated regions of the fibrous mats (to improve the acquisition of phosphorus peaks). SEM images of the spots where EDX analysis was conducted are presented inside the corresponding EDX spectrograms. The HAp-related calcium (Ca) and phosphorus (P) peaks detected in the EDX spectra of the different samples are highlighted in red.

Randomly aligned piezoelectric PVDF-TrFE fibers filled with HAp nanoparticles at different concentrations – 0%, 1%, 3%, 5% and 10% – were successfully generated via electrospinning, by previously optimized operational parameters and PVDF-TrFE concentration. Their morphology was assessed by SEM (Figure 1). As displayed in Figure 1(b), the obtained scaffolds exhibited average diameters within the range of 10–500 nm, which corresponds to the average diameter of the collagen fibrils found within the bone tissue [33] and that the fibers are intended to emulate: the produced PVDF-TrFE and PVDF-TrFE/HAp nanofibers had diameters ranging from 231 to 432 nm. Additionally, while a slight increase in fiber diameter appeared to occur with the increase in HAp concentration, no linear correlation between both parameters was observed. A homogenous distribution of HAp nanoparticles within the fibrous structure of the scaffolds was achieved for all experimental conditions (Figure 1(a)): this was confirmed by both the reduced presence of aggregates in the nanofibers and the detection of both calcium (Ca) and phosphorus (P) in the elemental composition analysis (EDX) of regions of the fibrous scaffolds with and without visible aggregates (SI, Figure S1). Overall, fluor (F), calcium (Ca) and phosphorus (P) were detected through EDX analysis of the different generated scaffolds, thus corroborating the presence of both PVDF-TrFE and HAp as part of the composition of the fibers (Figure 1(c)). The incorporation of HAp did not have any noticeable impact on the surface topography of the nanofibers, as AFM analysis showed similarly smooth-surfaced fibers for the scaffolds with the highest HAp concentrations (SI, Figure S2).

The contact angle of the piezoelectric nanofibers was measured to evaluate their degree of wettability, which constitutes an important feature for TE applications. All fibrous scaffolds were found to be hydrophobic given that contact angles above 90° were obtained for all experimental conditions (SI, Figure S3). Nevertheless, a slight decrease in the hydrophobicity of the PVDF-TrFE fibers was observed with the addition of HAp nanoparticles, even though no linear trend between HAp concentration and the contact angle of the resulting fibers was registered. In particular, a statistically significant decrease in contact angle was observed for the piezoelectric nanofibers with 1% and 5% HAp nanoparticles compared with the PVDF-TRFE-only fibers. These results appear to suggest that the addition of HAp nanoparticles to the scaffold composition could have a positive influence with respect to cell adhesion and proliferation.

**Figure 2.**
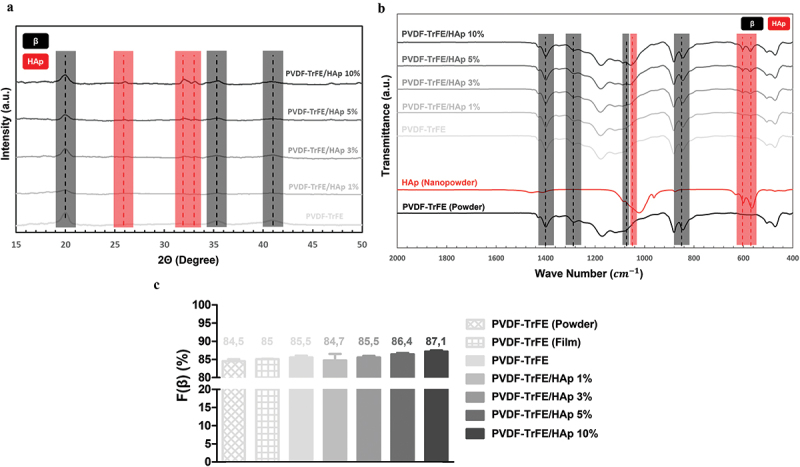
XRD (a) and FTIR (b) analysis of PVDF-TrFE powder, HAp nanopowder, and PVDF-TrFE and PVDF-TrFE/HAp fibers. Estimated relative β-phase content (%) (c) of PVDF-TrFE powder, PVDF-TrFE film, and PVDF-TrFE and PVDF-TrFE/HAp fibers. Three different samples (n=3) were used in the analysis.

The chemical properties of the piezoelectric scaffolds with varying concentrations of HAp were characterized by XRD and FTIR analysis (Figure 2). Three diffraction peaks attributed to PVDF-TrFE were identified at 2Θ values of 20°, 35.3° and 41° (assigned to the (110, 200), (001) and (201) lattice planes, respectively) in the collected XRD patterns of both non-functionalized and functionalized PVDF-TrFE nanofibers, therefore corroborating the presence of the PZP as part of the composition of the scaffolds (Figure 2(a)) [34]. The HAp-containing nanofibers had lower intensities of the PVDF-TrFE associated diffraction peaks than their non-functionalized counterpart (especially for the (001) and (201) lattice planes). This could be potentially explained by a reduced relative proportion of the PZP on the composition of the PVDF-TrFE/HAp nanofibers due to HAp addition. Three additional HAp-related diffraction peaks – at 2Θ value of 25.9°, 31.9° and 33° (assigned to the (002), (211) and (112) lattice planes, respectively) – were detected in the XRD patterns of the composite PVDF-TrFE/HAp scaffolds, confirming the presence of the bioceramic nanoparticles (Figure 2(a)) [35]. As expected, an increase in the intensity of the respective diffraction peaks attributed to HAp could be observed with the increase in HAp nanoparticle concentration. Overall, the presence of broad diffraction peaks in the collected XRD data, as opposed to the narrower peaks found in crystalline structures, appears to suggest that these fibers present a more amorphous architecture.

Further confirmation of the presence of both PVDF-TrFE and HAp as part of the composition of the composite scaffolds was obtained through FTIR analysis (Figure 2(b)). For all experimental conditions, major IR peaks attributed to the β crystalline phase of PVDF-TrFE, which has an all-trans planar-zigzag chain conformation and is responsible for the polymer’s piezoelectric features, were identified in the spectra, namely at ca. 850 cm^−1^ (-CF_2_ symmetric stretching), 1073 cm^−1^ (-CH_2_ wagging, C–C stretching), 1288 cm^−1^ (-CF_2_ symmetric stretching, -CH_2_ scissoring, C–C stretching) and 1400 cm^−1^ (-CH_2_ wagging, C–C stretching) [36–38]. On the other hand, characteristic peaks of α phase, namely at 765, 976 and 1207 cm^−1^ [38], were extremely weak. This is in line with the reported tendency of PVDF-TrFE to preferentially form a crystalline phase similar to the ferroelectric all-trans β phase of PVDF, and the lack of non-polar crystalline phases (α phase) in PVDF-TrFE copolymers [38]. Moreover, three IR peaks assigned to an important functional group of HAp (phosphate group) were detected on the FTIR spectra of the composite fibers, therefore corroborating the presence of the bioceramic on the structure of those scaffolds: 570 cm^−1^ and 604 cm^−1^ (P-O asymmetric bending vibration of (PO_4_)^3-^ groups), and 1049 cm^−1^ ((PO_4_)^3-^ asymmetric stretching) [39,40]. IR peaks corresponding to other HAp-related functional groups were also identified, even if they were not as intense as those from (PO_4_)^3-^, namely at ca. 1419 and 1453 cm^−1^ (C-O asymmetric stretching vibration) and at 875 cm^−1^ (asymmetric bending in CO_3_^2-^ groups). An increase in the intensity of the HAp related IR peaks was observed with the increase in HAp concentration in the fibers.

### 3.2. Effect of HAp concentration on the piezoelectric and piezoresistive properties of electrospun nanofibers

The β-phase content of the fibers was estimated to indirectly assess the impact of HAp concentration on the piezoelectric properties of the nanofibers, given the crucial role of this crystalline phase on the electroactive features of the PZP. These estimations were performed applying Equation (1). As observed in Figure 2(c), there was a slight increase in the β-phase content of the fibers with the increase in HAp concentration. This suggests a favorable impact of HAp addition on the piezoelectric properties of PVDF-TrFE fibers. Higher β-phase content values were also obtained for the PVDF-TrFE electrospun fibers compared with both the PVDF-TrFE powder (lowest value) and PVDF-TrFE films. This suggests that the electrospinning of PVDF-TrFE also improves its piezoelectricity as a result of the combined electrical poling and mechanical stretching of the charged jet that is ejected during electrospinning [41–43].

Further confirmation of the role of HAp on the overall piezoelectric properties of the as-spun PVDF-TrFE fibers was provided by estimating the scaffolds’ β-phase content through the collected XRD patterns (Equation (2)). As can be shown in Table S1 (SI), the PVDF-TrFE/HAp nanofibers had higher β-phase content when compared with the PVDF-TrFE scaffolds, further supporting the beneficial role that HAp played in shifting the molecular structure of PVDF-TrFE to an all-trans conformation. However, the linear increase in β-phase content with the increase in HAp nanoparticle concentration, which was observed with the FTIR data treatment (Equation (1)), was not replicated with the XRD patterns (SI, Tables S1 and S2).

**Figure 3.**
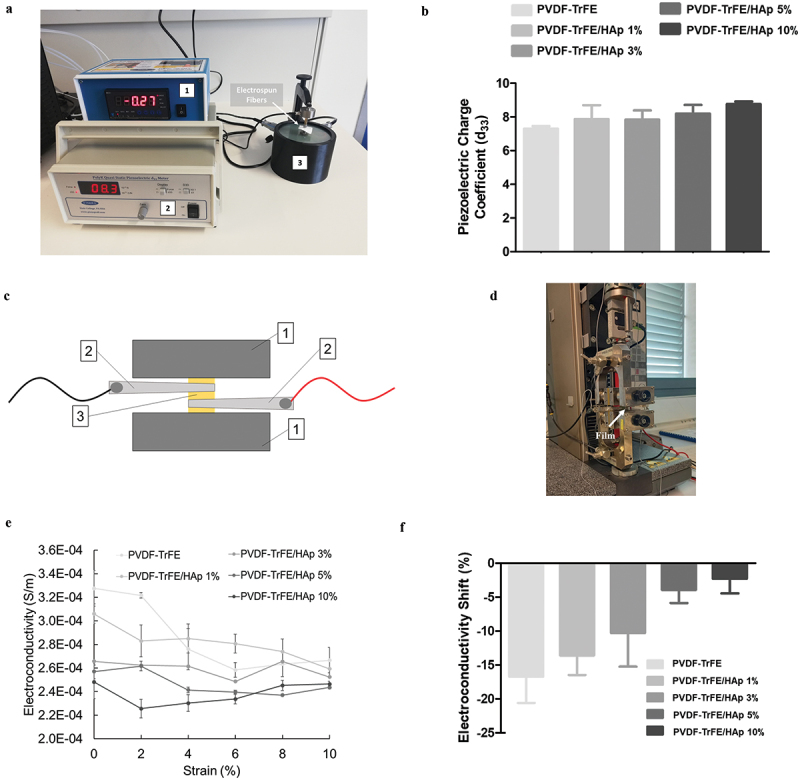
Photo (a) of the setup used for the quasi-static measurement of the piezoelectric charge coefficient (d_33_) of the as-spun PVDF-TrFE nanofibers with and without HAp. 1 – static force sensor; 2 – Berlincourt piezoelectric meter; 3 – piezoelectric sensor. Piezoelectric charge coefficient (d_33_) (b) of the PVDF-TrFE nanofibers with and without HAp. Scheme (c) and photo (d) of the setup used for the connection of the PVDF-TrFE/HAp films to a multimeter using metallic clips, while a sample was being held by the clamps of a tensile testing instrument used for deformation. 1 – clamps of tensile testing equipment; 2 – metallic clips connected to multimeter; 3 – PVDF-TrFE/HAp film. Variation of electroconductivity (S/m) with deformation for PVDF-TrFE/HAp films (e). Electroconductivity shifts (f) obtained for the different films between the 0% and 10% strain measurement points. Three different measurements per condition (n=3) were used in the analysis.

The piezoelectric charge coefficient (d_33_) of the PVDF-TrFE-based scaffolds was measured using a quasi-static piezoelectric meter in order to further corroborate the role of HAp on the piezoelectric properties of the nanofibers. As shown in Figure 3(b), an increase in the value of d_33_ of the scaffolds was observed with the addition of HAp (SI, Table S3). This means that, when exposed to similar mechanical loads, the nanofibers with higher HAp concentrations become more polarized, and, as a result, present enhanced piezoelectric features [28]. This result is aligned with prior observations regarding the β-phase content of the scaffolds.

As a strategy for further improving the piezoelectric properties of the generated composite scaffolds, the PVDF-TrFE fibers without HAp and with the highest HAp concentration (10%) were annealed according to two distinct procedures – P1 and P2 – that were adapted from the literature. As expected, a statistically significant increase in β-phase content was obtained for the annealed fibers compared with the as-spun scaffolds (SI, Table S2 and Figure S6), with similar results being registered for both annealing procedures.

The highest β-phase content values were registered for the annealed PVDF-TrFE fibers with 10% HAp: 93.5% and 92.2% for the P1 and P2 annealed scaffolds, respectively (SI, Table S2). These results appear to suggest that the combined addition of HAp to the formulation of the composite fibers as well as the annealing of PVDF-TrFE fibers is responsible for an increase in the piezoelectricity of the scaffolds. No significant structural/morphological shifts of the fibrous scaffolds were observed after annealing, with only a small increase in the average fiber diameter being registered for the P1 annealed scaffolds (SI, Figure S4). The same PVDF-TrFE β-phase and HAp-related IR peaks were identifiable on the FTIR spectra of the annealed fibers (SI, Figure S5).

The piezoresistivity of the generated as-spun nanofibers, i.e. their ability to shift their electrical resistivity as a result of an applied mechanical load, was confirmed indirectly by using a mechanical tester setup on films (with the same material composition) that were coupled to a digital multimeter. As demonstrated in Figure 3(e), a clear decrease in the electroconductivity of the films was observed with the increase in applied strain for all experimental conditions, therefore corroborating their piezoresistive properties. The increase in HAp concentration of the films appeared to be associated with a decreased piezoresistive effect, as smaller shifts in electroconductivity were observed for the films with the highest HAp concentrations (−18.6% and −0.7% electroconductivity shifts for the PVDF-TrFE vs PVDF-TrFE/HAp 10% films, respectively) (Figure 3(f)).

### 3.3. Effect of HAp concentration on the mechanical properties of piezoelectric electrospun nanofibers

**Figure 4.**
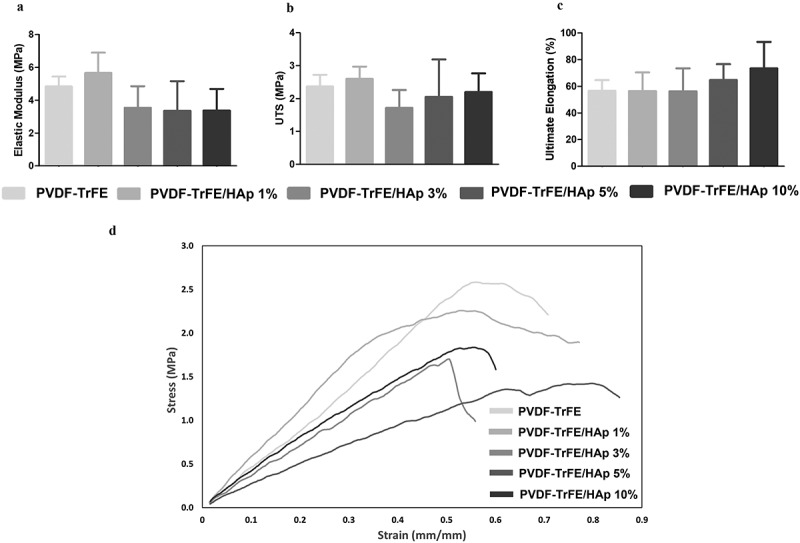
Mechanical properties of the as-spun PVDF-TrFE and PVDF-TrFE/HAp piezoelectric scaffolds obtained after tensile testing: elastic modulus (a), ultimate tensile strength (UTS) (b), ultimate elongation (c) and representative stress–strain curves (d). Five samples (n=5) were used in the analysis.

An analysis of the mechanical properties of the generated as-spun PVDF-TrFE and PVDF-TrFE/HAp scaffolds under tensile testing is presented in Figure 4. As demonstrated by the representative stress–strain curves shown in Figure 4(d), no drastic changes to the mechanical properties of the PVDF-TrFE nanofibers under tensile testing were observed with the incorporation of the HAp nanoparticles. A slight non-significant decrease in the elastic modulus of the piezoelectric scaffolds was registered with the increase in HAp concentration (Figure 4(a)). On the other hand, a moderate increase in the ultimate elongation was detected with the increase in HAp concentration in the composition of the fibers (Figure 4(c)). The values for ultimate tensile stress (UTS) of the scaffolds remained similar with the incorporation of the HAp nanoparticles (Figure 4(b)).

Meaningful shifts in the mechanical properties of the bioactive scaffolds were observed for the P1 and P2 annealed nanofibers (SI, Table S4 and Figure S7). A statistically significant increase in elastic modulus was obtained for the P1 annealed PVDF-TrFE fibers without HAp and for the fibers functionalized with 10% HAp. Indeed, after P1 annealing, the elastic modulus increased 2.28 folds (from 5 ± 1 MPa to 11 ± 3 MPa) and 4.30 folds (from 3 ± 1 MPa to 15 ± 4 MPa) for the PVDF-TrFE and PVDF-TrFE/HAp 10% scaffolds, respectively (SI, Table S4 and Figure S7). Smaller, but statistically significant, increases in the elastic modulus of the fibers were observed with the P2 annealed scaffolds, corresponding to increases of 2.07 folds (from 5 ± 1 MPa to 10 ± 3 MPa) and 3.04 folds (from 3 ± 1 MPa to 10 ± 2 MPa) for the fibers without HAp and with 10% HAp, respectively (SI, Table S4 and Figure S7).

A statistically significant decrease in the ultimate elongation was registered for the same set of annealed fibers. Indeed, after P1 annealing, the ultimate elongation decreased 4.94 folds (from 57 ± 8% to 11 ± 3%) and 6.57 folds (from 73 ± 20% to 11 ± 1%) for the nanofibers without HAp and with 10% HAp, respectively. Moreover, after P2 annealing, the ultimate elongation decreased 3.14 folds (from 57 ± 8% to 18 ± 3%) and 5.19 folds (from 73 ± 20% to 14 ± 5%) for the PVDF-TrFE and PVDF-TrFE/HAp 10% P2 annealed fibers, respectively (SI, Table S4 and Figure S7). A slight decrease in the UTS (non-statistically significant) was also obtained for both P1 and P2 annealed scaffolds (SI, Table S4 and Figure S7). Overall, although stiffer, the P1 and P2 annealed fibers were less prone to deformation and were more brittle when compared with the as-spun fibers, making them inadequate to develop scaffolds capable of properly mimicking weight-bearing tissues such as bone.

### 3.4. In vitro scaffold mineralization

The scaffolds were incubated in SBF at 37°C for a 28-day period in order to evaluate their ability to support the formation of a biologically appropriate mineral coating on their surface (osteoconductivity), critical for BTE applications.

**Figure 5.**
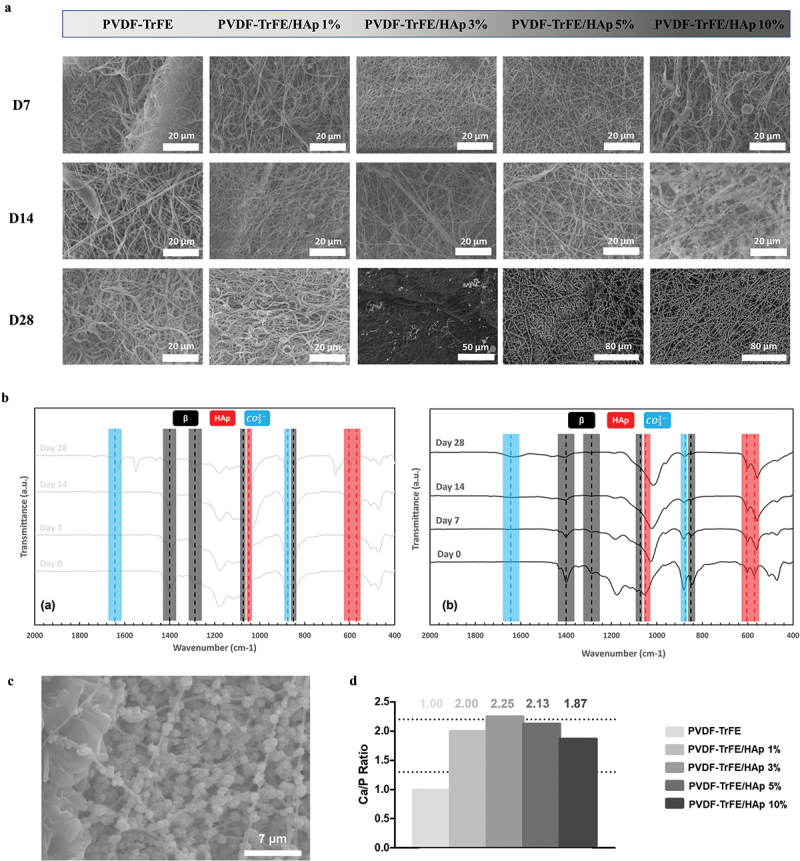
SEM micrographs (a) of the different mineralized fibrous piezoelectric scaffolds after 7 (D7), 14 (D14) and 28 (D28) days of incubation in SBF. FTIR (b) analysis of the PVDF-TrFE nanofibers – with 0% (a) and 10% (b) HAp – after 0, 7, 14 and 28 days of incubation in SBF. Close-up (c) of the mineralized PVDF-TrFE/HAp 10% fibers after 28 days of SBF incubation. Calcium/phosphorus (Ca/P) ratio (d) of the HAp crystal coating formed on the surface of the different piezoelectric scaffolds. The range of Ca/P ratios reported for human bone tissue is highlighted with dashed lines. Scale bar values are depicted in the SEM images.

The mineralization process was successful, as shown by Figure 5. After 7 days of incubation, no mineral coating appeared to form on the piezoelectric fibers’ surface, and no appreciable changes in the EDX and FTIR spectra were found (Figure 5(a,b)). However, at day 14 of incubation, it was evident that HAp-like constructs had formed, particularly on the PVDF-TrFE/HAp 10% scaffolds (Figure 5(a)). After 28 days of incubation, mineral crystals were clearly visible for the PVDF-TrFE scaffolds with 3%, 5% and 10% HAp (Figure 5(a–c)). After 14 days of incubation in SBF, HAp- and carbonate-group-related IR peaks (at ca. 570, 604 and 1049 cm^−1^ for HAp and 875 and 1644 cm^−1^ for $CO_{3}^{2-}$) were identified on the FTIR spectra of all piezoelectric scaffolds (Figure 5(b) and SI, Figure S8). While more noticeable increases in the intensity of those peaks were detected in the FTIR spectra of the control PVDF-TrFE scaffolds, smaller increases in intensity of the HAp-related IR peaks (masked effect) were observed for the PVDF-TrFE/HAp fibers. These results were further confirmed by EDX analysis, where calcium (Ca) and phosphorus (P) were detected in all samples (SI, Figure S9). Overall, the presence of HAp nanoparticles within the composition of the fibers appeared to contribute positively to the formation of mineral crystals on the surface of the respective scaffolds. Dispersed and heterogeneous mineral deposits were observed on the surface of the fibrous mats with lower HAp concentrations. On the contrary, full homogeneous single-fiber coatings were obtained for the scaffolds with the highest HAp nanoparticle concentrations (Figure 5(c)).

The calcium/phosphorus (Ca/P) ratio of the mineral coatings generated on the surface of the different piezoelectric fibers, after 28 days of mineralization, was estimated using the collected EDX data. As shown in Figure 5(d), the Ca/P ratios that were obtained for the different scaffolds were, for the most part, within the range of values reported for the human bone tissue (1.3–2.2) [44], with only the control fibers without HAp presenting a slightly lower ratio. While an increase in the atomic percentage of calcium (Ca) and phosphorus (P) in the mineralized PVDF-TrFE/HAp fibers was observed compared to their non-mineralized counterparts, their Ca/P ratios remained similar. This suggests that the mineral coatings formed on the surface of these scaffolds retained a Ca/P ratio similar to that of human bone tissue (SI, Figure S9).

### 3.5. Influence of HAp content of the piezoelectric electrospun scaffolds on MSC proliferation and osteogenic differentiation

In order to evaluate the effect of the generated piezoelectric PVDF-TrFE and PVDF-TrFE/HAp electrospun scaffolds on cell proliferation and osteogenic differentiation, hBM-MSCs were seeded on top of the fibrous scaffolds and cultured for 21 days under osteogenic medium.

**Figure 6.**
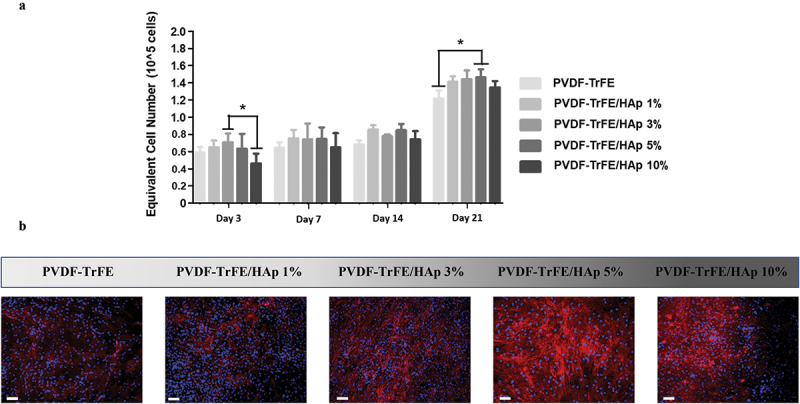
AlamarBlue cell metabolic activity assay (a) and DAPI/Phalloidin staining (b) of hBM-MSCs cultured on as-spun PVDF-TrFE and PVDF-TrFE/HAp piezoelectric scaffolds for 21 days. DAPI stains cell nuclei blue, while phalloidin stains actin-rich cytoskeleton red. Scale bar: 100 μm. Five different samples (n=5) were used in the cell proliferation assay analysis; *p < 0.05.

AlamarBlue assay was used to monitor MSC proliferation at days 3, 7, 14 and 21 of culture (Figure 6(a)). Cell growth was observed for all experimental conditions, with a notable spike in equivalent cell numbers being registered between days 14 and 21 of culture. Higher equivalent cell numbers and fold increases were obtained for the HAp nanoparticle-containing piezoelectric nanofibers, suggesting a beneficial effect of HAp on MSC growth. Statistically significant fold increases of 2.35±0.10, 2.40±0.17 and 2.44±0.15 were obtained at day 21 of cell culture (relative to day 0) for the PVDF-TrFE scaffolds with 1%, 3% and 5% HAp, respectively, when compared with the control PVDF-TrFE nanofibers (2.03±0.15) (SI, Table S5 and Figure S10). A notable statistically significant difference in equivalent cell numbers was also observed between the PVDF-TrFE/HAp 5% and PVDF-TrFE nanofibers at day 21 of culture.

DAPI/Phalloidin staining of the cells was also performed after 21 days of MSC osteogenic differentiation to evaluate cell morphology, adhesion and distribution across the different scaffold conditions (Figure 6(b)). A high density of cells could be observed on top of all piezoelectric scaffolds tested, especially for the PVDF-TrFE/HAp 1%, 3% and 5% fibers, indicating the successful adhesion and proliferation of the MSCs. The cells were also spread across the entire surface of the scaffolds and were stretched/aligned in different directions, which is likely related to the random orientation of the electrospun nanofibers.

**Figure 7.**
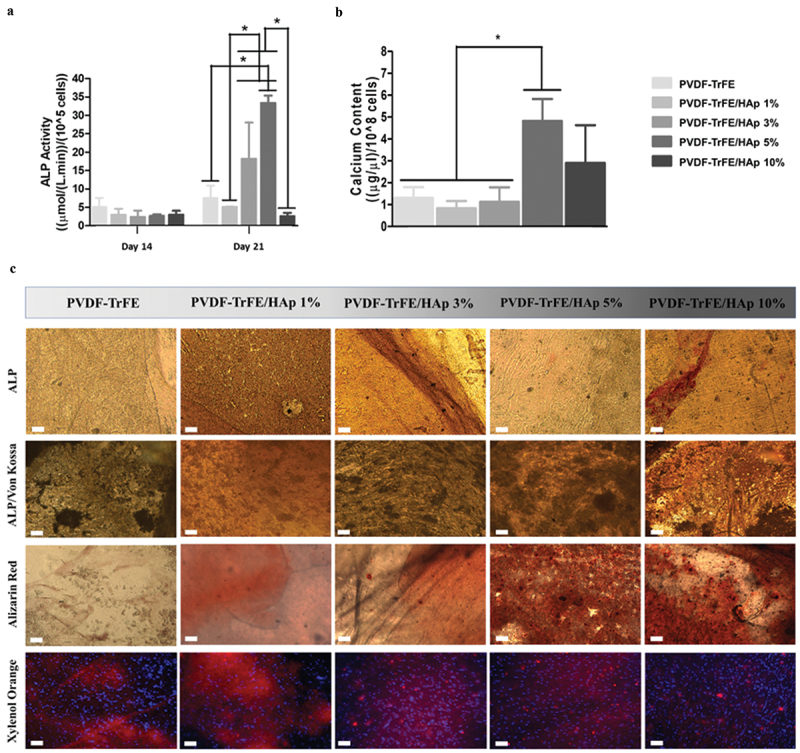
ALP activity (a) of MSCs seeded on PVDF-TrFE-based fibers after 14 and 21 days of osteogenic differentiation. Calcium content (b) secreted by MSCs cultured on PVDF-TrFE-based fibers after 21 days of osteogenic differentiation. ALP, ALP/Von Kossa, Alizarin Red and Xylenol Orange stainings (c) of hBM-MSCs differentiated on as-spun PVDF-TrFE and PVDF-TrFE/HAp piezoelectric scaffolds for 21 days. ALP staining stains ALP molecules red, while Von Kossa stains calcium deposits black. Alizarin Red stains calcium deposits red. Xylenol Orange fluorescent staining indicates the presence of calcium deposits in red. DAPI stains cell nuclei blue. Scale bar: 100 μm. Three different samples (n=3) were used in the analysis presented in (a) and (b); *p < 0.05.

The evaluation of osteogenic differentiation of MSCs cultured on the fibers was paramount to understand the potential applications of these scaffolds in BTE. Thus, the quantification of ALP activity, calcium deposition and complementary stainings were performed. After 14 days of culture in the osteogenic medium, no significant differences in ALP activity were identified between the fibrous scaffolds with and without HAp nanoparticles (Figure 7(a)). Statistically significant increases in ALP activity were registered after 21 days of culture for the PVDF-TrFE/HAp 3% and 5% nanofibers. A more moderate increase in ALP activity was observed, after 21 days culture, for the PVDF-TrFE and PVDF-TrFE/HAp 1% scaffolds, whereas the PVDF-TrFE/HAp 10% fibers exhibited no meaningful ALP activity increase between days 14 and 21 of osteogenic differentiation (Figure 7(a)). A statistically significant difference in the value of calcium content was observed for the PVDF-TrFE/HAp 5% fibers when compared with the control PVDF-TrFE, PVDF-TrFE/HAp 1% and 3% nanofiber scaffolds (Figure 7(b)). Relatively high calcium content levels (but non-significant) were also obtained for the PVDF-TrFE/HAp 10% scaffolds (Figure 7(b)).

Osteogenic stainings (ALP/Von Kossa, Alizarin Red and Xylenol Orange) were performed after 21 days of osteogenic differentiation to evaluate the obtained cell-secreted ECM (Figure 7(c)). ALP/Von Kossa staining corroborated ALP activity in all scaffolds (red markings) as well as the formation of cell-derived calcium deposits (darker regions), thus demonstrating the successful differentiation of MSCs into pre-osteoblasts for all experimental conditions. Alizarin Red and fluorescent Xylenol Orange stainings further confirmed the presence of calcium deposits (red staining) in all scaffolds. Despite PVDF-TrFE/HAp samples producing more intense stainings when compared with the control PVDF-TrFE-only fibers, no clear differences between the different PVDF-TrFE/HAp conditions could be identified.

**Figure 8.**
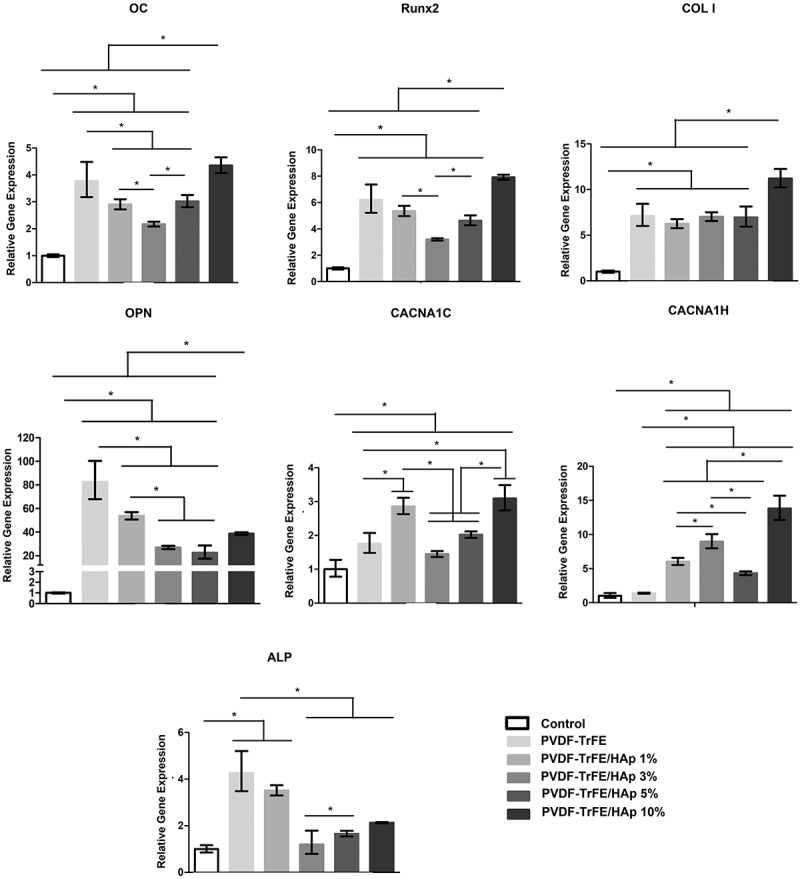
RT-qPCR analysis of hBM-MSCs cultured on the different piezoelectric scaffolds after 21 days of osteogenic differentiation. Expressions of target genes *ALP*, *Runx2*, *COL I*, *OC*, *OPN*, *CACNA1C* and *CACNA1H* were normalized to *GADPH* (housekeeping gene) and calculated as fold-change relative to the baseline expression of the control sample (hBMSC before scaffold seeding at day 0). Three different samples (n=3) were used in the RT-qPCR analysis; *p < 0.05.

RT-qPCR analysis of samples collected after 21 days of culture was used to assess the expression of key osteogenic marker genes associated with various stages of osteogenic differentiation, including *ALP*, *Runx2*, type I collagen (*COL I*), osteocalcin (*OC*) and osteopontin (*OPN*) (Figure 8). A significantly up-regulated expression of *ALP*, *Runx2*, *COL I*, *OC* and *OPN* was observed for all tested scaffolds compared with the control condition (hBM-MSCs at day 0). A statistically significant increase in *Runx2*, *COL I* and *OC* gene expression was detected for the MSCs seeded on PVDF-TrFE/HAp 10% scaffolds compared with the PVDF-TrFE and PVDF-TrFE/HAp nanofibers with lower HAp concentrations (1%, 3% and 5%). No significant differences in *COL I* expression were identified between PVDF-TrFE and PVDF-TrFE/HAp 1%, 3% and 5% nanofibers. However, *Runx2* and *OC* were down-regulated for the PVDF-TrFE/HAp 1%, 3% and 5% scaffolds. The cells seeded on the control PVDF-TrFE samples displayed an increased expression of *ALP* and *OPN* (statistically significant) when compared with the PVDF-TrFE/HAp samples. A decreasing trend in the expression of these marker genes (*ALP* and *OPN*) was observed with the increase in HAp concentration present in the nanofibers.

The gene expressions of *CACNA1C* and *CACNA1H* – which encode for the α-1C and α-1 H subunits of type L and type T voltage-gated calcium channels (VGCCs), respectively – were also assessed by RT-qPCR analysis to indirectly assess the impact of piezoelectric self-stimulation (triggered by MSC adhesion and migration in the piezoelectric substrate) on the osteogenic differentiation process [45,46]. VGCCs have been found to play a critical role in the piezoelectric stimulation of cells: stress-mediated piezoelectric stimuli are responsible for destabilizing the membrane potential of cells (hyperpolarization), which, in turn, causes the opening of VGCCs, leading to an influx of calcium ions [46]. This augmented calcium intake by the cells is then responsible for triggering intracellular calcineurin and calmodulin pathways, which directly impact gene expression and promote the production of key bone-related growth and differentiation factors (e.g. BMP-2) [47]. As shown in Figure 8, an up-regulated expression of both *CACNA1C* and *CACNA1H* was obtained for all tested scaffolds compared to the control condition. A significantly increased expression of the VGCCs was also detected for the MSCs cultured on the HAp-containing scaffolds when compared with the control PVDF-TrFE nanofibers. In particular, a statistically significant increase in *CACNA1C* and *CACNA1H* gene expression was obtained for the MSCs seeded on the PVDF-TrFE/HAp 10% nanofibers compared to the PVDF-TrFE and PVDF-TrFE scaffolds with lower HAp content, with only the PVDF-TrFE/HAp 1% scaffolds displaying similar, albeit slightly lower, *CACNA1C* gene expression.

**Figure 9.**
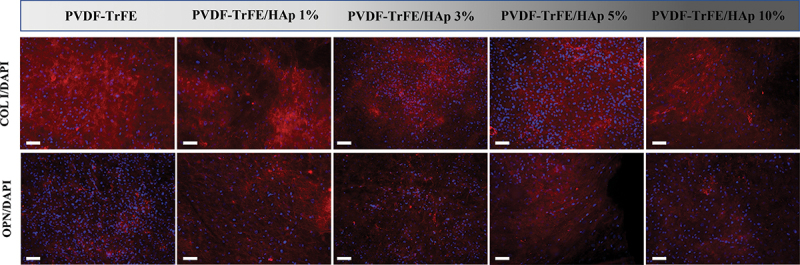
Immunofluorescence analysis to evaluate the presence of type I collagen (COL I) and osteopontin (OPN) on the electrospun scaffolds, after 21 days of osteogenic differentiation. The samples were counterstained with DAPI. DAPI stains cell nuclei blue, while COL I and OPN antibodies stain collagen and osteopontin molecules red, respectively. Scale bar: 100 μm.

Immunofluorescence staining of the different piezoelectric scaffolds after 21 days of culture was performed to assess the presence of the key bone ECM proteins type I collagen and osteopontin within the tissue constructs formed. As shown in Figure 9, both type I collagen and osteopontin were identified for all experimental conditions, with no clear differences in marker intensity being observed.

**Figure 10.**
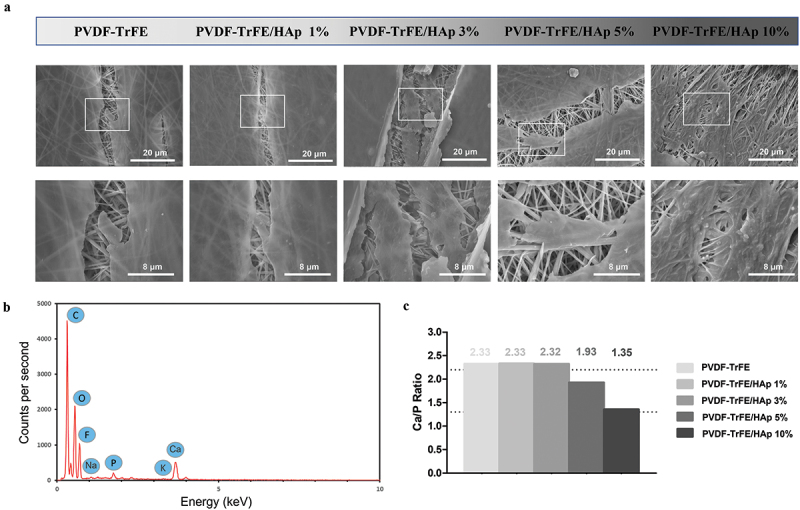
SEM micrographs (a) of the different cell-seeded composite piezoelectric nanofibers after 21 days of osteogenic differentiation: close-up (bottom) of regions of the cell-seeded scaffolds highlighted on top. Elemental composition analysis (b) of cell-seeded PVDF-TrFE nanofibers without HAp after 21 days of cell culture. EDX spectra were collected on non-coated regions of the fibrous mats (to improve acquisition of phosphorus peaks). Calcium/phosphorus (Ca/P) ratio (c) of the cell-derived mineral deposits formed on the surface of the different piezoelectric scaffolds.

The morphology of the cells and composition profile of the generated mineral deposits at day 21 of culture were evaluated through SEM imaging and elemental composition analysis. As shown in Figure 10(a), all piezoelectric scaffolds were found to be densely populated by differentiated cells. Through EDX analysis, cell-derived mineralization was further confirmed for all experimental conditions with the identification of calcium (Ca) and potassium (K) deposits, as well as phosphorus (P), sodium (Na) and, to a lesser extent, magnesium (Mg), which are commonly found in the inorganic phase of bone ECM (SI, Figure S11). As an example, an EDX spectra collected for the control PVDF-TrFE fibers is provided in Figure 10(b). The Ca/P ratio of the cell-derived calcium deposits was close to the range of values found in the native bone tissue (Figure 10(c)).

## 4. Discussion

The strategy of merging known native tissue electrical properties in combination with exogenous electrical stimulation is often overlooked in the TE field. Therefore, there is significant potential for developing smart piezoelectric material-based scaffolds for BTE applications. Such scaffolds would make it possible to directly provide piezoelectric stimulation to injured areas, potentially speeding up regeneration, without the need for implanted electrodes (e.g. ultrasound), while also recapitulating a significant electrical property present in native bone tissue. As a result, novel piezoelectric and piezoresistive PVDF-TrFE/HAp electrospun nanofibers were developed and assessed in this study, envisaging their potential use in future BTE applications. Some limitations exist with using electrospinning for BTE, particularly, the reduced pore size of electrospun scaffolds with small-sized nanofibers, which negatively impact cell migration and tissue integration [19]. This limitation can, however, be addressed with different strategies, such as optimizing the operational parameters to generate fibers with larger diameters or producing sacrificial nanofibers through co-electrospinning [19].

Multiple studies have reported the development of random and aligned electrospun PVDF-TrFE nanofibers for BTE and other TE applications (e.g. cardiac TE) given the high piezoelectric coefficient and easy processability of this PZP [43,48]. This polymer constitutes a great alternative to other piezoceramics, which share a high piezoelectric coefficient, but are significantly hindered by their cytotoxicity. Damaraju *et al.* (2017) reported the development of randomly aligned heat-treated and as-spun PVDF-TrFE fibers that were able to promote the selective osteogenic and chondrogenic differentiation, respectively, of seeded MSCs under regular piezoelectric stimulation [49]. In a different study, Wang *et al.* (2019) developed aligned PVDF-TrFE fibers using a rotating drum collector, which supported an increased proliferation of mouse pre-osteoblasts [50]. In the present study, to improve the osteoinductive potential of the fibrous PVDF-TrFE scaffolds, synthetic HAp nanoparticles were incorporated within the structure of the fibers to emulate the inorganic phase of native bone ECM. Multiple HAp concentrations (0–10%) were trialed to assess the effect of the nanoparticles on the physicochemical properties and osteogenic potential of the resulting fibrous scaffolds. A comparable approach was taken by Tandon *et al.* (2019), which fabricated HAp filled PVDF nanofibers with two distinct HAp concentrations (5% and 10%) and performed a physicochemical characterization of the generated scaffolds without addressing their biocompatibility [51]. More recently, dos Santos *et al.* (2022) developed similar PVDF/HAp nanofibers with a wider range of HAp nanoparticle concentrations (0–20%) [52]. By using PVDF instead of PVDF-TrFE, however, the piezoelectricity of the resulting scaffolds is expected to be lower, as this PZP comprises an intrinsically higher α-phase and reduced β-phase content (more thermodynamically favorable) comparatively to its derivative PVDF-TrFE. Additionally, scaffolds made of PVDF are frequently submitted to post-processing techniques (e.g. annealing, electrical poling) for improving their piezoelectric properties, which can negatively impact other properties of the scaffolds, namely their mechanical behavior. Such post-processing strategies for incrementing the β-phase content and, therefore, making the material piezoelectric, are not required for PVDF-TrFE-based scaffolds.

### 4.1. PVDF-TrFE/HAp nanofibrous scaffold characterization

Overall, the generated PVDF-TrFE/HAp fibers exhibited a smooth surface and a reduced number of visible mineral aggregates spread across the fibrous mats. The residual aggregate presence increased with the increase in HAp concentration. The homogeneous distribution of HAp nanoparticles along the structure of the fibers was confirmed through EDX analysis, corroborating, therefore, the appropriate functionalization of the PVDF-TrFE scaffolds. The average diameter of the fibers obtained in this study was within the range of diameters of the collagen fibrils present in the native bone tissue [33], with a slight increase in diameter being registered with the addition of HAp. This is in line with what was reported by Tandon *et al.* (2019) for the PVDF fibers with 5% and 10% HAp [51]. A similar phenomenon was observed by Lopresti *et al.* (2020) and Wutticharoenmongkol *et al.* (2006) with HAp nanoparticles containing PLA and polycaprolactone (PCL) electrospun nanofibers, respectively [53,54].

The addition of HAp to the PVDF-TrFE-based casting solutions was found to slightly decrease the contact angle of the hydrophobic PVDF-TrFE nanofibers. This could be attributed in part to the significant presence of hydrophilic hydroxyl and phosphate groups within the structure of the nanoparticles. The opposite effect was observed by Tandon *et al.* (2019) with the PVDF/HAp electrospun scaffolds [51]. Such a discrepancy could be potentially related to important differences between the chemical structures of PVDF and PVDF-TrFE and, ultimately, to the way both PZPs interact with HAp. The results obtained here are in line with those obtained for other HAp functionalized biomaterials, including polyurethane nanofibers (also hydrophobic) functionalized with HAp, as reported by Nahavandizadeh *et al.* (2020) [55]. Moreover, other studies have also registered an improvement in the wettability of PVDF films with the addition of HAp [56,57].

Fiber composition was analyzed through XRD and FTIR analysis. Both diffraction and IR peaks assigned to PVDF-TrFE and HAp were identified on the collected XRD and FTIR data, therefore corroborating the composite nature of the generated nanofibers. As expected, an increase in the intensity of the HAp-related diffraction and IR peaks was observed with the increase in nanoparticle concentration. Through the collected XRD data it was also possible to assess a reduction in scaffold crystallinity, as a result of the addition of HAp, which is in accordance with previous reports [51].

The detection of β-PVDF-TrFE related IR peaks in the FTIR spectra corroborated the piezoelectric nature of the composite PVDF-TrFE/HAp fibers. The β-phase content of the different fibrous scaffolds was estimated to indirectly evaluate the impact of HAp concentration on the overall piezoelectricity of the electrospun nanofibers. A slight increase in β-phase content of the fibrous scaffolds was obtained with the increase in HAp concentration, from which we can infer that the ceramic is responsible for promoting a shift in the molecular conformation of PVDF-TrFE to an all-trans configuration (β-phase nucleation), and therefore incrementing the piezoelectricity of the PZP. We hypothesize that the combined effect of high electronegativity of the fluoride and hydrogen atoms in PVDF-TrFE and the ionic nature of HAp is responsible for promoting such molecular configuration. Similar behavior was described with other PVDF nanofillers (e.g. barium titanate, carbon nanotubes), where the interaction of negatively charged fillers with the positively charged -CH_2_ groups or the interaction of positively charged nanofillers with the negatively charged -CF_2_ groups of PVDF was the underlying cause for the shift in the molecular conformation of the PZP [43,58]. In particular, a number of studies have reported an increase in β-phase content by combining calcium-containing nanofillers (e.g. calcium-doped zinc oxide, CaCO_3_) and PVDF [59,60]. These results were further confirmed by estimating the β-phase content of the fibers using the collected XRD patterns. Once again, higher β-phase content values were obtained for the HAp-containing scaffolds. On the contrary, both Tandon *et al.* (2019) and dos Santos *et al.* (2022) reported a decrease in β-phase content of the PVDF fibers with the addition of HAp [51,52]. This disparity in results may once again be attributed to differences in the interaction of the HAp nanoparticles with the carrier PZPs, being likely related to the reduced polarity of PVDF (more prevalent non-polar α phase) comparatively to its derivative PVDF-TrFE. Quasi-static piezoelectric charge coefficient (d_33_) measurements of the as-spun PVDF-TrFE fibers with and without HAp further supported the hypothesis of a positive influence of the bioceramic on the overall piezoelectric features of the resulting scaffolds, as a clear increase in d_33_ was observed with the increase in HAp concentration in the fibers. In a study performed by Gorodzha *et al.* (2017), a similar increase in d_33_ was observed by adding silicate-containing hydroxyapatite (Si-HAp) to poly(hydroxybutyrate-co-hydroxyvalerate) (PHBV) nanofibers [61]. The piezoelectric charge coefficient values obtained for the different PVDF-TrFE/HAp scaffolds were lower than those reported in some studies with electrospun PVDF-TrFE nanofibers [62,63]. It should also be noted that a number of studies have reported values of d_33_ close to zero for non-poled PVDF-TrFE electrospun scaffolds [50,64]. This discrepancy in results is likely related to differences in the parameters used during the measurement of the piezoelectric charge coefficient of the materials, from the frequency and static force applied to the thickness of the samples introduced in the sensor, a phenomenon which is particularly exacerbated for soft materials, such as electrospun fibrous meshes [65].

Statistically significant increases in β-phase content were obtained for the generated PVDF-TrFE and PVDF-TrFE/HAp 10% fibers after annealing. This observation is in line with previous studies, which report that by heating the scaffolds at temperatures slightly above the temperature of α-phase relaxation (70–85°C) it is possible to achieve the rearrangement of the chains of the PZP (β-phase nucleation) without negatively impacting its crystalline structure and, as a result, improve the piezoelectric properties of the material [26,66].

The piezoresistivity of the scaffolds was corroborated by observing shifts in the electroconductivity of the films (with identical composition) while applying increasing strain. Smaller changes in electroconductivity were registered for the films with higher HAp content compared with the control PVDF-TrFE films. This might be explained by the presence of HAp clusters (more predominant in films with higher HAp concentrations), which may be responsible for disrupting the intrinsic piezoresistive features of PVDF-TrFE. The decreasing trend of electroconductivity of the films with the increase in applied strain could also be correlated with an increase in amorphous phase of PVDF-TrFE with stretching in a similar phenomenon to what was described by Sun *et al.* (2010) with PVDF films [67]. As expected, lower electroconductivity values were measured for the HAp-containing PVDF-TrFE films, which is likely the result of the nanoparticles’ resistivity. Piezoresistive materials have significant potential in TE, given that they can be applied as wearable and stretchable strain sensors for healthcare monitoring (e.g. pulse/respiration monitoring, drug delivery monitoring) and human motion detection, or as components of pressure sensors for detection of subtle pressures (e.g. blood flow) [68,69]. A number of studies have reported the use of PVDF and PVDF-TrFE in the development of piezoresistive sensors with potential applications in TE [70–72].

The functionalization of the fibrous PVDF-TrFE scaffolds did not significantly impact the mechanical properties of the resulting electrospun nanofibers, as only a slight decrease in elastic modulus and an increase in ultimate elongation were observed with the addition of HAp nanoparticles (non-statistically significant). While no studies on the impact of adding HAp to the mechanical properties of PVDF and PVDF-TrFE electrospun fibers under tensile testing can be found in the literature, the mixing of HAp nanopowder with other biomaterials, such as polyurethane and silk fibroin, has been mostly found to augment the stiffness of electrospun fibers [55,73,74]. Similar to what was observed in our study, Tong *et al.* (2010) reported unvarying Young’s modulus values with the addition of carbonated HAp nanoparticles to PHBV nanofibers [75]. Fiber annealing was found to significantly impact the mechanical properties of the as-spun fibers, with a moderate Young’s modulus increase and an expressive reduction of the ultimate elongation of the as-spun fibers under tensile testing being observed. This is in line with what was reported by Lam *et al.* (2019) for annealed PVDF-TrFE nanofibers [25]. Overall, the mechanical properties obtained under tensile testing for both the as-spun and annealed non-functionalized PVDF-TrFE nanofibers, were consistent with those reported elsewhere [25,51]. These results are also similar to the mechanical properties of native type I collagen fibrils (wet), which were described by Yang *et al.* (2008) as presenting a shear modulus of 2.9±0.3 MPa [76].

Successful *in vitro* mineralization was achieved for both PVDF-TrFE and PVDF-TrFE/HAp nanofibers by incubating the scaffolds in SBF for 28 days. These results appear to suggest that the obtained piezoelectric fibers would be capable of being naturally mineralized when placed in an *in vivo* setting (*in vivo* bone bioactivity), which constitutes an important scaffold feature for replacing damaged bone tissue [31]. By generating mineral coatings with biomimetic Ca/P ratios *in vitro*, it is also expected that these implantable fibrous scaffolds would be capable of supporting the *in vivo* formation of HAp-like constructs chemically similar to those found in the inorganic phase of bone ECM. Apatite formation was significantly more homogeneous and comprehensive for the HAp-containing nanofibers. A similar result was reported by Malherbi *et al.* (2022) with HAp and β-tricalcium phosphate (β-TCP)-filled PVDF films (HAβ-PVDF) [77]. This may be explained by an increased polarity of the PZP as a result of β-phase nucleation (due to HAp addition), which is responsible for shifting the local environment of SBF and facilitating the recruitment of Ca^2+^ and PO_4_^3-^ ions found in the saline solution that, in turn, are absorbed and adhere to the surface of the nanofibers forming apatite-like structures [77].

### 4.2. Influence of HAp content on MSC proliferation and osteogenic differentiation

The effect of the piezoelectric PVDF-TrFE/HAp scaffolds on hBM-MSCs proliferation and osteogenic differentiation was evaluated for a 21-day culture period in osteogenic medium under static conditions. Through DAPI/Phalloidin staining and SEM imaging, we were able to confirm that a high density of cells had spread across the entire surface of all scaffolds, demonstrating the biocompatibility of the composite fibers, which is in line with what has been reported so far in the literature for the PZP and bioceramic materials used [52,56,78,79]. A more expressive cell growth was observed for the HAp-containing nanofibers, therefore corroborating their improved bioactivity. These results are in accordance with what has been described in several other studies for HAp filled nanofibers. Spadaccio *et al.* (2009) reported increased bioactivity for HAp nanopowder functionalized PLLA fibers [80]. In a different study, Thien *et al.* (2013) observed an augmented hBM-MSC growth with chitosan/HAp electrospun scaffolds [81]. More recently, Stastna *et al.* (2022) described the role of HAp-reinforced PCL nanofibers in promoting the proliferation of mouse fibroblasts [82].

The commitment of the hBM-MSCs towards the osteogenic lineage was confirmed for all the experimental groups with the detection of calcium deposits, confirming cell-derived mineralization, and ALP activity, an important precursor in bone ECM formation. Overall, higher ALP activity and calcium deposition were registered for the PVDF-TrFE/HAp nanofibers, once more highlighting their improved bioactivity and the important biological cues provided by the HAp nanoparticles present in the scaffold composition. Soheilmoghaddam *et al.* (2020) reported similar results as they also observed a more expressive ALP activity for MSC-seeded HAp-filled poly lactic-co-glycolic acid (PLGA) electrospun fibers [83]. While higher calcium content levels were observed for the PVDF-TrFE/HAp scaffolds with the highest HAp concentrations (5% and 10%), low ALP activity values were obtained for the PVDF-TrFE/HAp 10% nanofibers (contrasting with the high ALP activity values registered for the PVDF-TrFE/HAp 5% scaffolds). This result may be explained by the fact that ALP production is more predominant in the bone ECM production and initial maturation stages of osteogenic development that precede the tissue’s mineralization, after which its activity decreases [84,85]. As a result, it is possible that a faster differentiation of the MSCs cultured on the PVDF-TrFE/HAp 10% nanofibers occurred, motivated by the increased HAp nanoparticle presence, and, because of this, a lower ALP activity level was measured at day 21 of culture given the more advanced stage of maturation of the MSC-derived osteoblasts present on these scaffolds (mineralization stage). In essence, we hypothesize that while an ALP activity peak may have occurred between the days 14 and 21 of cell culture for the PVDF-TrFE/HAp 10% fibers, at the 21-day mark, its activity was already heavily impaired due to increased osteoblast maturity. This theory appears to be supported by the statistically significant up-regulation of *COL I* and *OC* gene expression by the cells present on these scaffolds after 21 days of culture, which are relevant biomarkers produced by mature osteoblasts at later stages of osteogenic development. The increased expression of *Runx2* on the HAp-containing scaffolds as well as the high expression of both *ALP* and *OPN* on the non-functionalized PVDF-TrFE nanofibers appear to suggest that cells with different degrees of maturation are present on the piezoelectric nanofibers. In agreement with our result, Peng *et al.* (2012) reported an up-regulation of *COL I*, *Runx2* and *OC* gene expression in HAp-filled chitosan electrospun fibers [86]. Moreover, our observations are also supported by the results from Liang *et al.* (2022), which described an augmented expression of *COL I* in BM-MSCs cultured on HAp/poly lactic acid (PLA) fibrous scaffolds [87]. All tested conditions stained positive for both *COL I* and *OPN*, which appeared to be evenly distributed within the structure of the different piezoelectric scaffolds. This is in line with what was described by Soheilmoghaddam *et al.* (2020) with PLGA/HAp nanofibers seeded with hBM-MSCs [83]. Damaraju *et al.* (2017) also reported positive staining for *COL I* with hBM-MSC-seeded PVDF scaffolds [49]. The up-regulated gene expressions of *CACNA1C* and *CACNA1H* by the cells on the PVDF-TrFE/HAp scaffolds, compared with the control PVDF-TrFE nanofibers, appear to suggest that the MSCs that were cultured on the functionalized scaffolds were subjected to a more significant piezoelectric self-stimulation. This could be potentially attributed to the increased piezoelectricity of the HAp-containing scaffolds, which have been previously found to present higher values of piezoelectric charge coefficient. Other studies have also reported an increased expression of VGCCs by cells seeded on piezoelectric biomaterials. In a study performed by Gouveia *et al.* (2017), an increased expression of *CACNA1C*, among other key ion channel subunits, was registered for rat cardiac cells seeded on magnetic PCL nanofilms coated with piezoelectric PVDF-TrFE microfibers compared to cells cultured on tissue culture polystyrene [88]. More recently, Kong *et al.* (2021) reported an up-regulated expression of *CACNA1C* and *CACNA1H* by macrophages seeded on β-PVDF scaffolds [89]. Camarero-Espinosa *et al.* (2021) observed an increased gene expression of *CACNA1C* by human BM-MSCs seeded on 3D printed Janus scaffolds comprised of PCL and PLA [90]. Interestingly, Camarero-Espinosa *et al.* were able to identify a correlation between the activity of type L VGCCs (of which *CACNA1C* is a component) and the osteogenic differentiation of MSCs, as they verified that, by blocking the activity of those ionic channels, a down regulation of *COL I*, *Runx2* and *OC* gene expression occurred [90]. This might explain why an up-regulated expression of the same set of osteogenic markers (*COL I*, *Runx2* and *OC*) was obtained for the MSCs seeded on the PVDF-TrFE/HAp scaffolds, which also happened to exhibit an increased expression of *CACNA1C*. Altogether, these results appear to suggest that the improved piezoelectric features of the HAp-containing scaffolds may have been responsible for an increase in piezoelectric self-stimuli by the seeded MSCs (and consequent increase in VGCC activity), which might have led to the apparent accelerated osteogenic differentiation process that was observed for the scaffolds with higher HAp concentrations.

An analysis of the cellular-derived mineral deposits formed on the surface of the fibers after 21 days of osteogenic differentiation was performed to further assess the osteogenic potential of the piezoelectric fibrous scaffolds: both calcium and potassium deposits were identified by elemental composition analysis, with other important elements such as sodium and magnesium, commonly found in bone ECM, being detected. Cell-derived apatite-like structures formed on the surface of all scaffolds were found to present biomimetic Ca/P ratios similar to the native bone tissue [44]. These results are consistent with those reported in past studies. Damaraju *et al.* (2013) reported the formation of mineral deposits with a bone-like Ca/P ratio (1.14) by BM-MSCs seeded on PVDF electrospun nanofibers [91]. In a different study, Marino *et al.* (2010) described a similar phenomenon with MSCs seeded on β-TCP (HAp-like mineral structure) scaffolds (Ca/P ratio of 1.63±0.03) [92].

Overall, these results appear to corroborate the osteogenic potential of the developed biomimetic HAp-containing piezoelectric nanofibers, highlighting their promising potential for future BTE applications. Moreover, the exploration of wireless electrical stimulation strategies through the mechanical stimulation of these piezoelectric scaffolds (either ultrasound-based [93] or exercise-induced [94]) might further enhance the regenerative outcomes, paving the way for novel less invasive, comfortable and more efficient clinical methods to treat bone defects. In vivo validation of the generated electroactive scaffolds using an animal model (possibly combined with external mechanical stimulation such as US) will also be considered in the future to assess the piezoelectric scaffolds’ performance (e.g. immunological response, scaffold integration with surrounding bone tissue) in a relevant whole-organism context.

## 5. Conclusions

In summary, we have successfully fabricated and characterized HAp-filled osteoinductive and piezoelectric PVDF-TrFE nanofibers. These scaffolds could emulate the fibrous nature and nanoscale dimensions of the collagen fibrils found in bone ECM as well as the native piezoelectricity of the tissue, while also retaining the mechanical features of the synthetic material and improving its piezoelectric and surface properties. Our results showed that HAp incorporation facilitated apatite formation on the surface of the scaffolds, therefore demonstrating their enhanced bioactivity. A higher MSC growth and an improved osteogenic performance were obtained for the PVDF-TrFE/HAp nanofibers, with an augmented ALP activity, cell-derived calcium deposition and bone-related gene expression being observed with the addition of HAp. Therefore, this work presents the combination of HAp nanoparticles and piezoelectric electrospun PVDF-TrFE fibers as a promising strategy for not only enhancing the piezoelectric properties and surface features of the electroactive scaffolds but also for improving their osteoinductive potential. Future studies on the effect of piezoelectric stimulation of MSC-seeded PVDF-TrFE/HAp scaffolds in culture should be conducted to further investigate the potential role of piezoelectricity in promoting bone tissue regeneration.

## Supplementary Material

Supplemental Material
