# Supplementary material for: Hydroxyapatite-filled osteoinductive and piezoelectric nanofibers for bone tissue engineering
Source: Sci Technol Adv Mater. 2023 Aug 24;24(1):2242242. doi: 10.1080/14686996.2023.2242242 (PMC10453998; doi:10.1080/14686996.2023.2242242)
Supplement: Supplemental Material [file TSTA_A_2242242_SM1899.docx]

**Supplementary Information**

**Hydroxyapatite-filled osteoinductive and piezoelectric nanofibers for bone tissue engineering**

Frederico Barbosa^1,2^, Fábio F. F. Garrudo^1,2,3^, Paola S. Alberte^1,2^, Leonor Resina^1,2,4^, Marta S. Carvalho^1,2^, Akhil Jain^5^ , Ana C. Marques^6^, Francesc Estrany^4^, Frankie J. Rawson^5^, Carlos Aléman^4,7^, Frederico Castelo Ferreira^1,2*^, João C. Silva^1,2*^

^1^ Department of Bioengineering and iBB-Institute for Bioengineering and Biosciences, Instituto Superior Técnico, Universidade de Lisboa, Av. Rovisco Pais, 1049-001 Lisboa, Portugal

^2^ Associate Laboratory i4HB – Institute for Health and Bioeconomy, Instituto Superior Técnico, Universidade de Lisboa, Av. Rovisco Pais, 1049-001 Lisboa, Portugal

^3^ Department of Bioengineering and Instituto de Telecomunicações, Instituto Superior Técnico, Universidade de Lisboa, Av. Rovisco Pais, 1049-001 Lisboa, Portugal

^4^ Departament d’Enginyeria Química and Barcelona Research Center for Multiscale Science and Engineering, EEBE, Universitat Politècnica de Catalunya, Barcelona 08019, Spain

^5^ Bioelectronics Laboratory, Regenerative Medicine and Cellular Therapies, School of Pharmacy, Biodiscovery Institute, University of Nottingham, Nottingham, NG7 2RD, UK

^6^ CERENA, Department of Chemical Engineering, Instituto Superior Técnico, Universidade de Lisboa, Av. Rovisco Pais, 1049-001 Lisboa, Portugal

^7^ Institute for Bioengineering of Catalonia (IBEC), The Barcelona Institute of Science and Technology, Baldiri Reixac 10-12, 08028 Barcelona, Spain

*Corresponding authors: Dr. João Carlos Silva, e-mail: [joao.f.da.silva@tecnico.ulisboa.pt](mailto:frederico.ferreira@tecnico.ulisboa.pt)

Prof. Frederico Castelo Ferreira, e-mail: [frederico.ferreira@tecnico.ulisboa.pt](mailto:frederico.ferreira@tecnico.ulisboa.pt)

Manuscript prepared for ***Science and Technology of Advanced Materials***, June 2023

Morphological and Physicochemical Properties of PVDF-TrFE/HAp Nanofibers


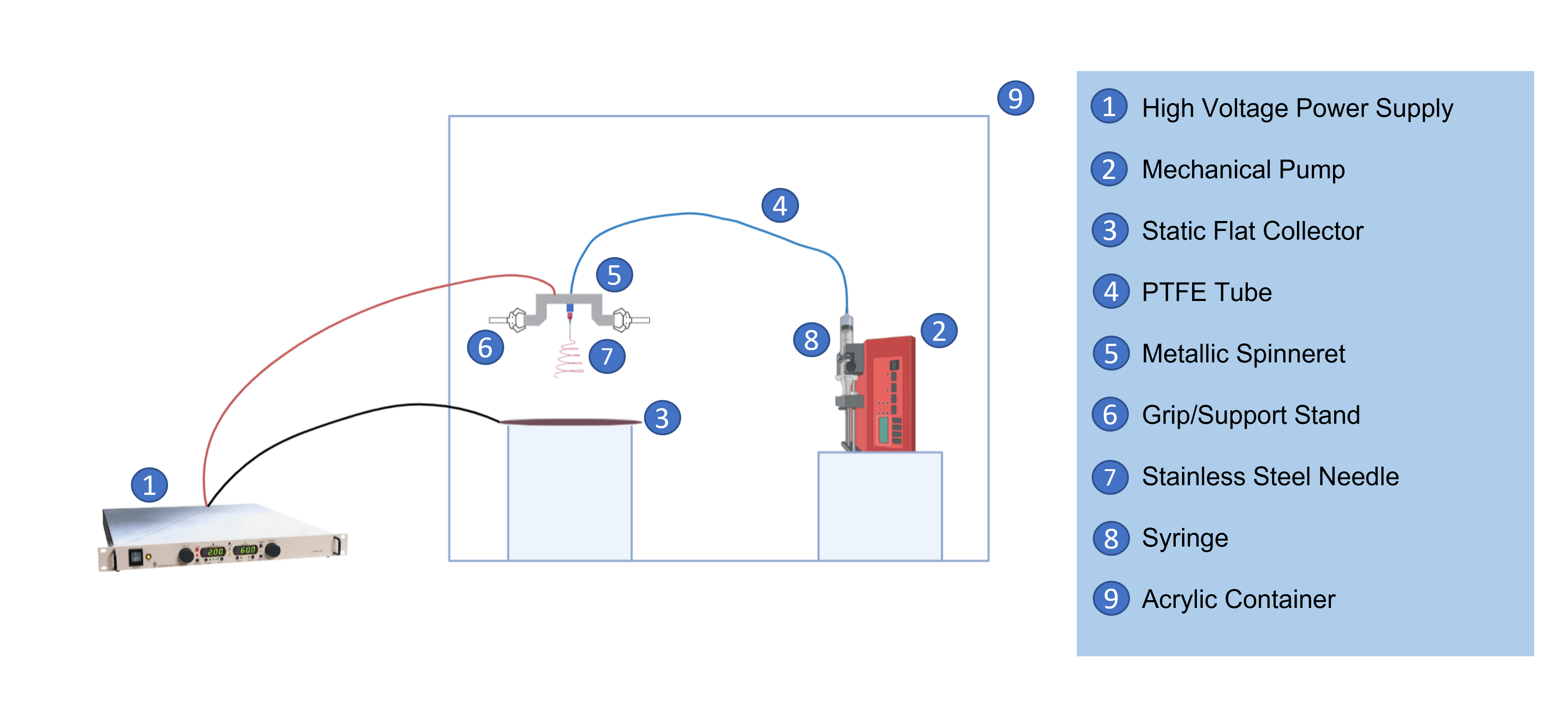


**Scheme S1.** Simplified scheme of the electrospinning setup used.


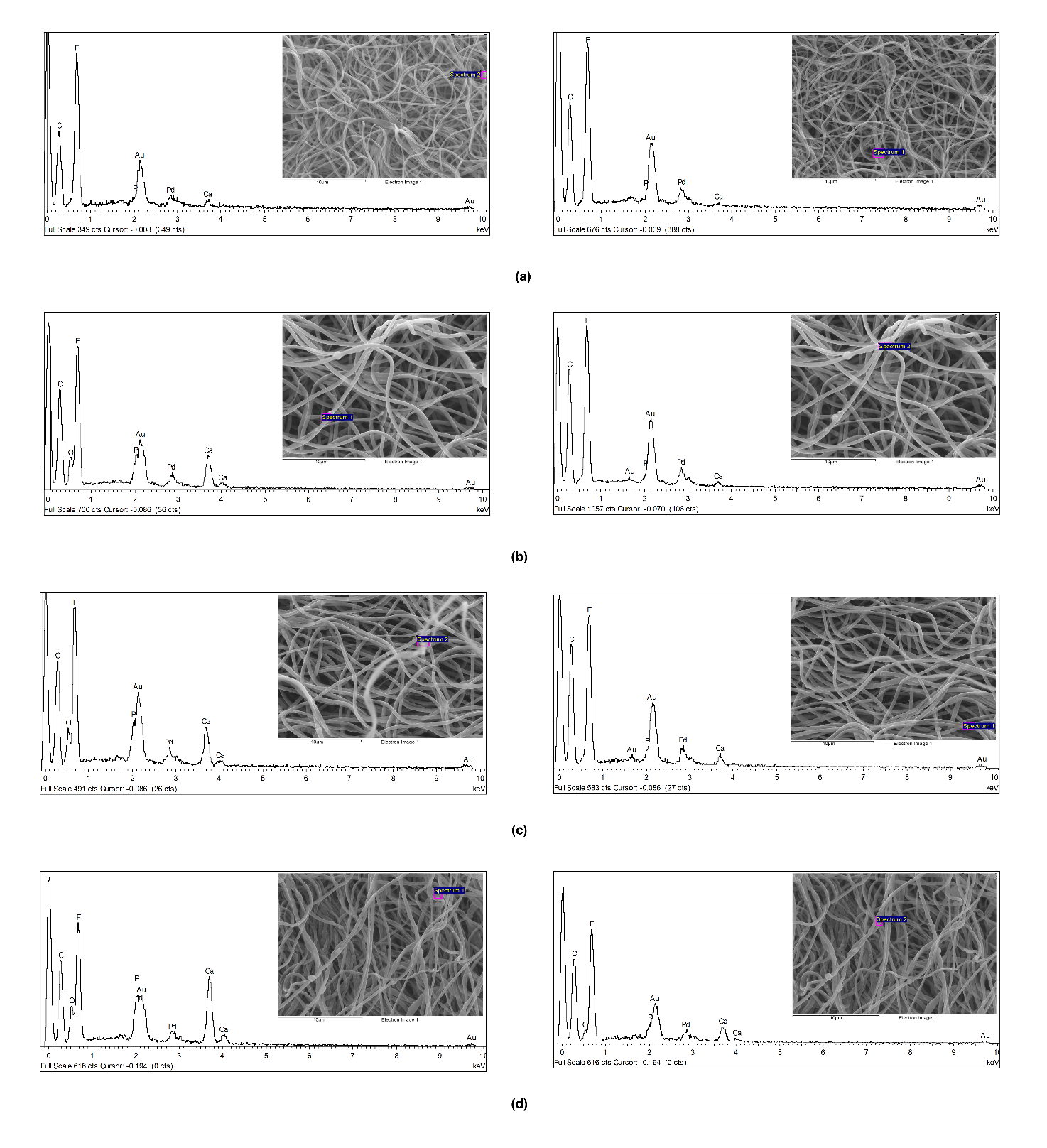
**Fig S1.** Elemental composition analysis (EDX) of as-spun PVDF-TrFE/HAp nanofibers: PVDF-TrFE fibers functionalized with 1% (a), 3% (b), 5% (c) and 10% (d) HAp. EDX spectrograms were collected in regions with aggregates (left) and without visible aggregates (right) of the fibrous mats. SEM images of the spots were EDX analysis was performed are presented inside the corresponding EDX spectrogram.

**
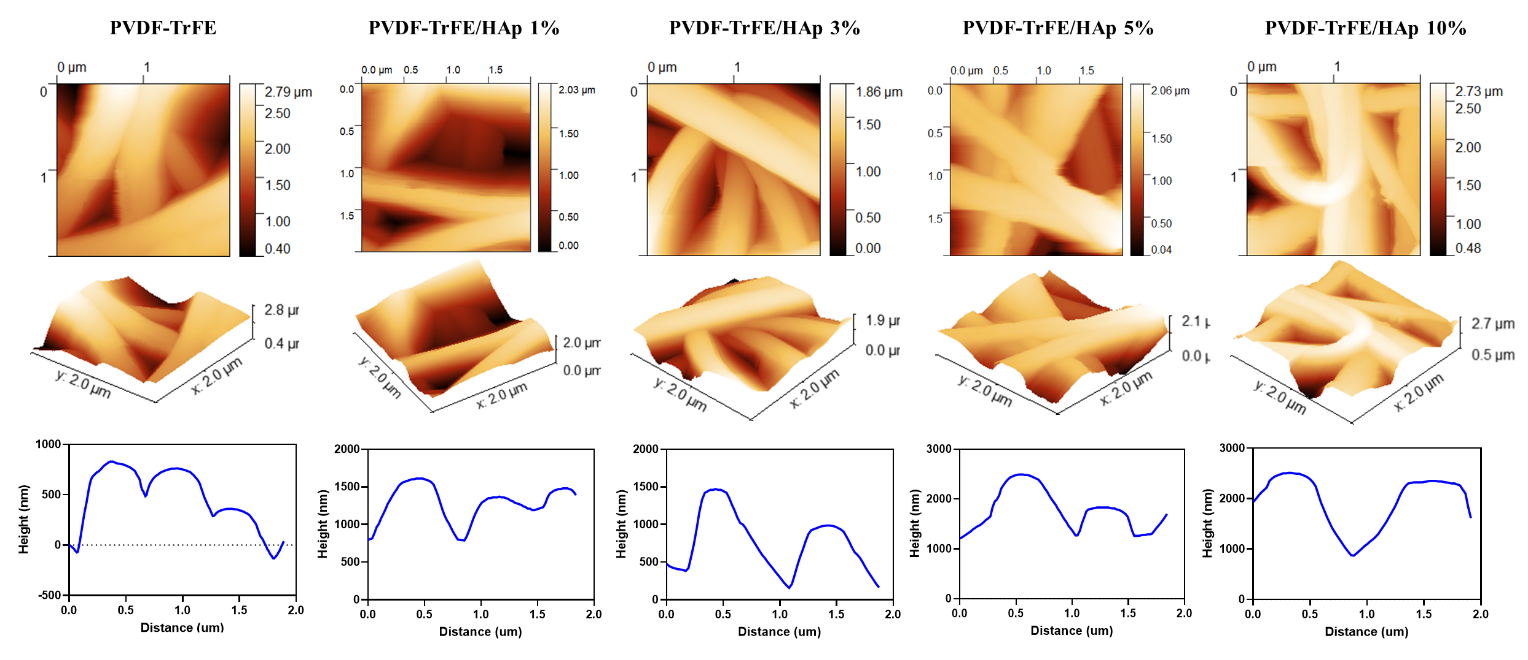
**

**
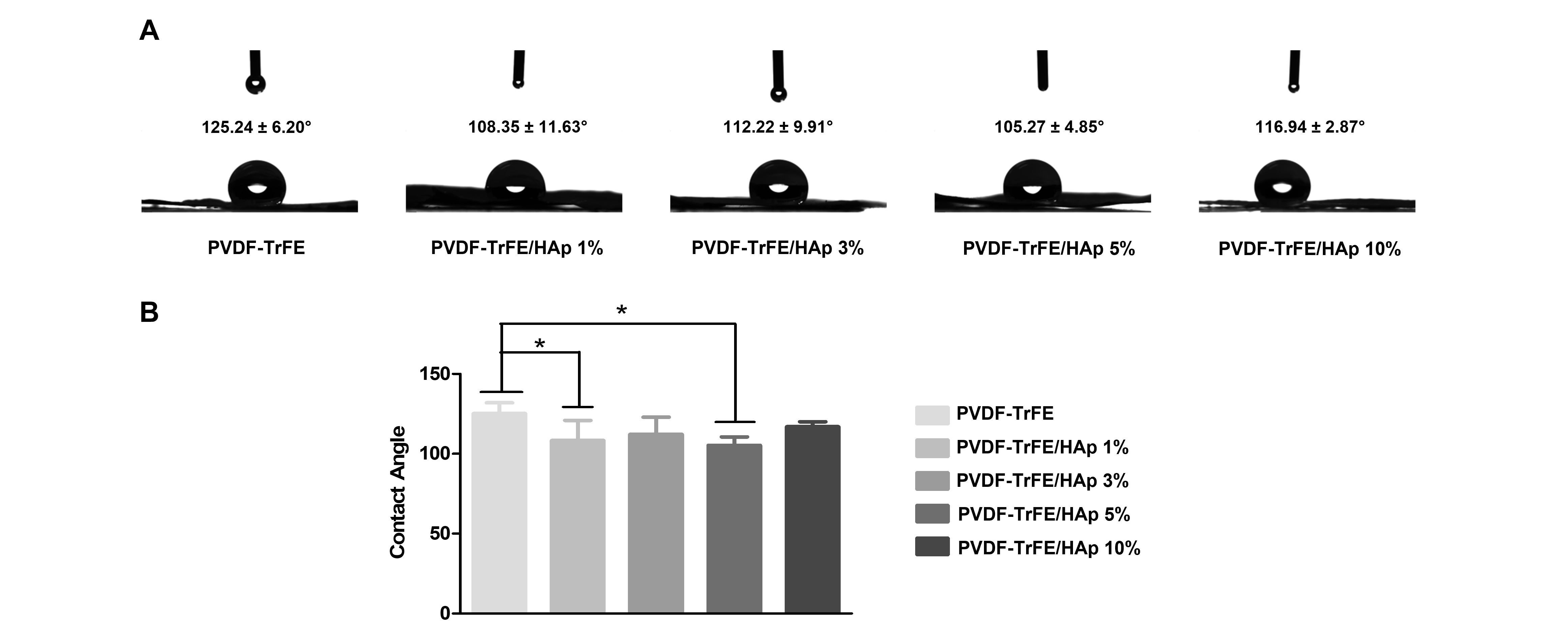
Fig S2.** Atomic Force Microscopy (AFM) analysis of the different as-spun PVDF-TrFE/HAp nanofibers: 2D AFM image (top), 3D AFM image (middle) and 2D profile (bottom) of the scaffolds.

Fig S3. Sessile drop droplet profile (A) and contact angle (B) of the as-spun PVDF-TrFE and PVDF-TrFE/HAp fibers. Seven different samples ($\boldsymbol{n=7}$) were used in the analysis; *p < 0.05.

Piezoelectric Properties of PVDF-TrFE/HAp Nanofibers: Effects of Fiber Annealing

**Table S1.** Summary of XRD-based estimated relative β phase content (%) of as-spun PVDF-TrFE and PVDF-TrFE/HAp fibers. A single sample per experimental condition (n = 1) was used in the analysis.

|  | **Relative β-Phase Content (%)** |
| --- | --- |
| PVDF-TrFE Fibers | 27.81 |
| PVDF-TrFE/HAp 1% | 49.13 |
| PVDF-TrFE/HAp 3% | 32.11 |
| PVDF-TrFE/HAp 5% | 47.17 |
| PVDF-TrFE/HAp 10% | 36.21 |

**
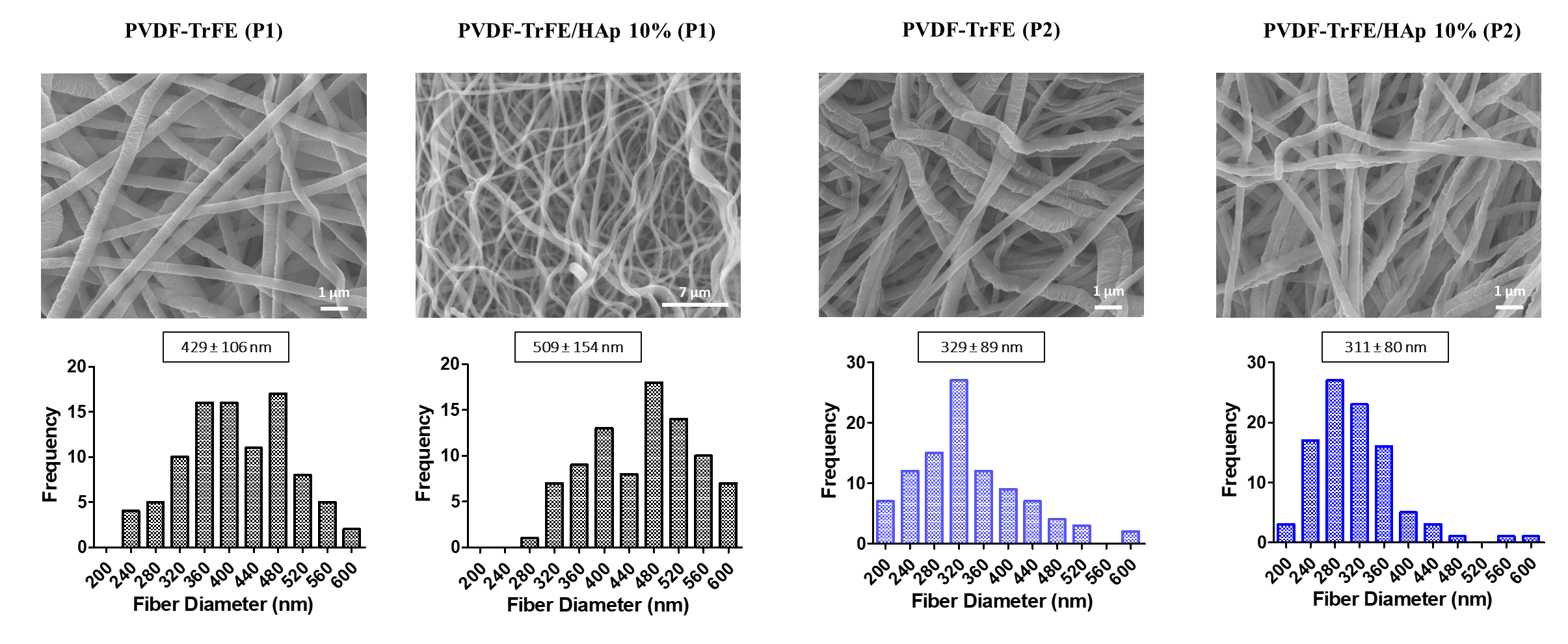
**

**Fig S4.** SEM images (top) and fiber diameter distribution histograms (bottom) of the annealed PVDF-TrFE and PVDF-TrFE/HAp 10% fibers. Scale bar values are depicted in the images.

**
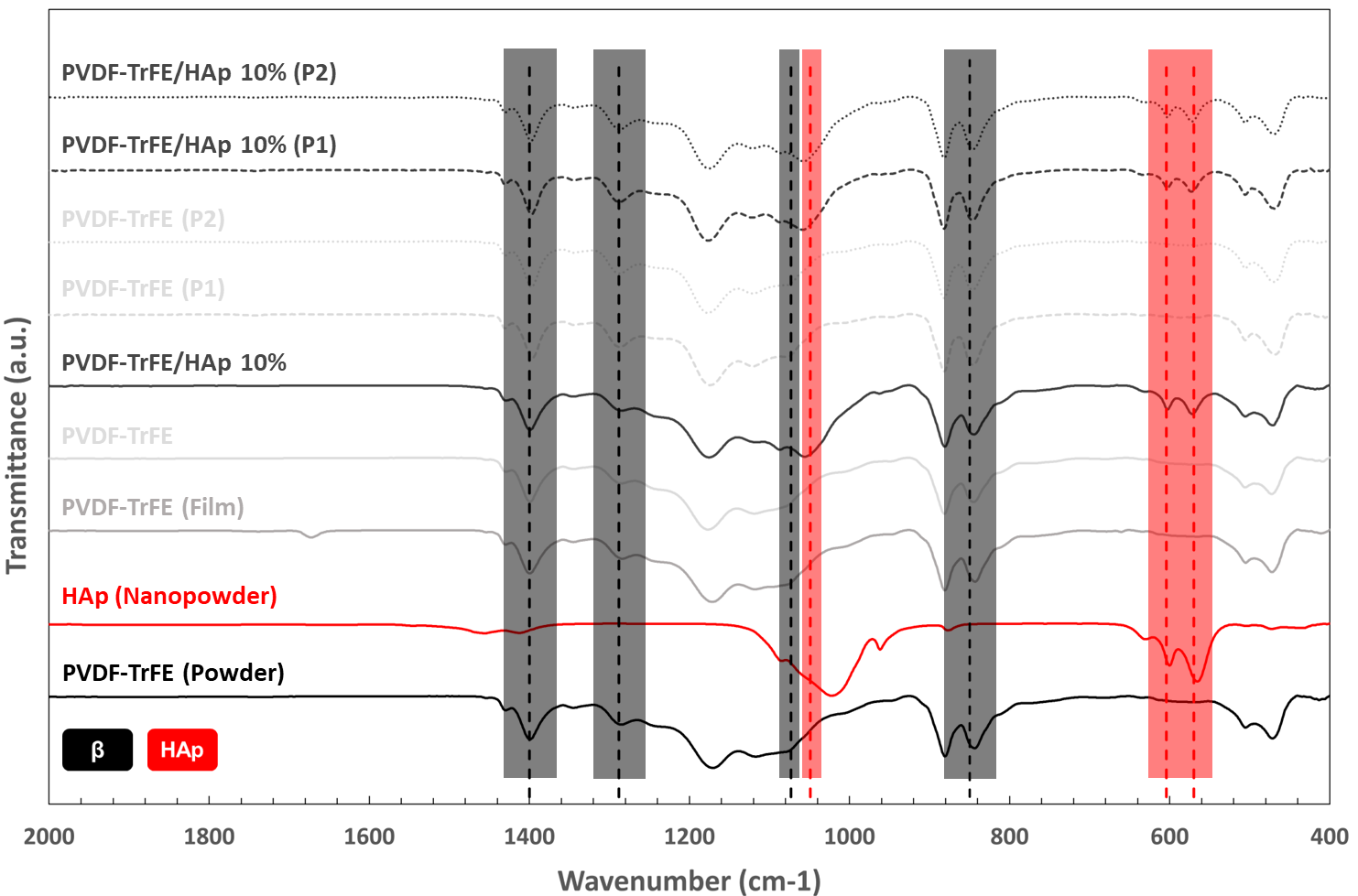
**

**Fig S5.** FTIR analysis of PVDF-TrFE powder, HAp nanopowder, PVDF-TrFE films, and as-spun and annealed PVDF-TrFE based fibers without and with HAp (0% and 10%, wt%). Four characteristic PVDF-TrFE β-phase related IR peaks (850, 1073, 1288 and 1400 cm^−1^) were identified on the graphic. Additionally, three characteristic HAp related IR peaks (570, 604 and 1049 cm^−1^) were also highlighted.

**Table S2.** Summary of FTIR-based estimated relative β phase content (%) of PVDF-TrFE powder, PVDF-TrFE films, and as-spun and annealed PVDF-TrFE and functionalized PVDF-TrFE/HAp fibers. Three different samples (n = 3) were used in the analysis.

|  | **Relative β-Phase Content (%)** |
| --- | --- |
| PVDF-TrFE Powder | 84.49 ± 0.55 |
| PVDF-TrFE Film | 85.07 ± 0.10 |
| PVDF-TrFE Fibers | 85.53 ± 0.50 |
| PVDF-TrFE/HAp 1% | 84.70 ± 1.82 |
| PVDF-TrFE/HAp 3% | 85.52 ± 0.45 |
| PVDF-TrFE/HAp 5% | 86.39 ± 0.36 |
| PVDF-TrFE/HAp 10% | 87.14 ± 0.35 |
| PVDF-TrFE Fibers (P1) | 92.85 ± 1.24 |
| PVDF-TrFE Fibers (P2) | 92.16 ± 1.33 |
| PVDF-TrFE/HAp 10% (P1) | 93.50 ± 1.68 |
| PVDF-TrFE/HAp 10% (P2) | 92.17 ± 0.52 |


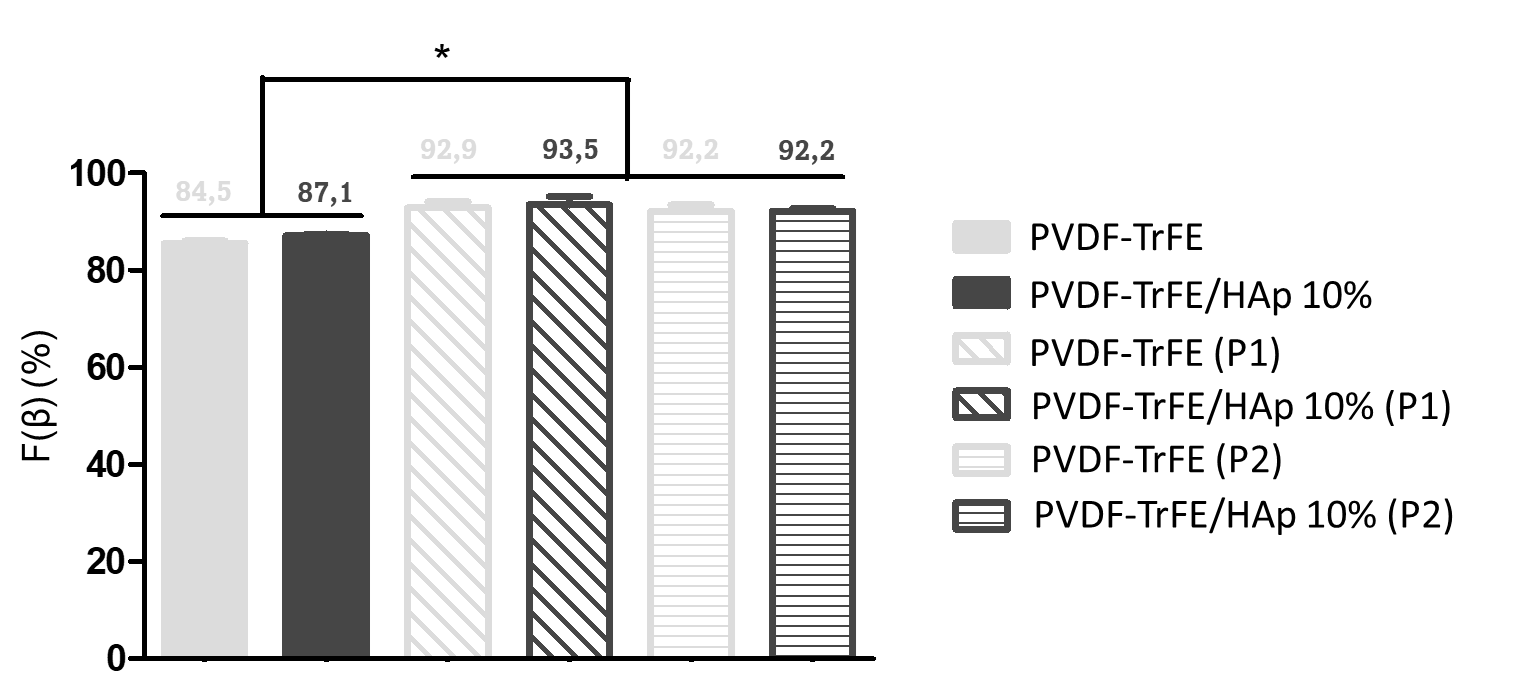


**Fig S6.** FTIR-based estimated relative β phase content (%) of as-spun and annealed PVDF-TrFE and PVDF-TrFE/HAp 10% fibers. Three different samples (n=3) were used in the analysis; *p < 0.05.

**Table S3.** Piezoelectric charge coefficient (d_33_) values measured for the as-spun PVDF-TrFE nanofibers with and without HAp. Three different samples (n = 3) were used in the analysis.

|  | **d_33_ (pC/N)** |
| --- | --- |
| PVDF-TrFE | 7.30 ± 0.26 |
| PVDF-TrFE/HAp 1% | 7.87 ± 1.43 |
| PVDF-TrFE/HAp 3% | 7.83 ± 0.96 |
| PVDF-TrFE/HAp 5% | 8.20 ± 0.89 |
| PVDF-TrFE/HAp 10% | 8.77 ± 0.25 |

Mechanical Properties of PVDF-TrFE/HAp Nanofibers

**Table S4.** Mechanical properties of as-spun and annealed PVDF-TrFE and PVDF-TrFE/HAp piezoelectric scaffolds obtained after tensile testing: elastic modulus, ultimate tensile strength (UTS) and ultimate elongation. Five different samples (n = 5) were used in the analysis.

|  | **Elastic Modulus (MPa)** | **UTS**  **(MPa)** | **Ultimate Elongation (%)** |
| --- | --- | --- | --- |
| PVDF-TrFE | 4.85 ± 0.60 | 2.37 ± 0.36 | 56.66 ± 7.98 |
| PVDF-TrFE/HAp 1% | 5.66 ± 1.24 | 2.60 ± 0.37 | 56.46 ± 14.01 |
| PVDF-TrFE/HAp 3% | 3.54 ± 1.31 | 1.71 ± 0.54 | 56.27 ± 17.26 |
| PVDF-TrFE/HAp 5% | 3.36 ± 1.80 | 2.05 ± 1.14 | 64.68 ± 11.95 |
| PVDF-TrFE/HAp 10% | 3.38 ± 1.32 | 2.20 ± 0.57 | 73.50 ± 19.90 |
| PVDF-TrFE (P1) | 11.05 ± 3.42 | 1.34 ± 0.65 | 11.48 ± 3.20 |
| PVDF-TrFE/HAp 10% (P1) | 14.55 ± 3.63 | 1.85 ± 0.48 | 11.19 ± 0.88 |
| PVDF-TrFE (P2) | 10.04 ± 3.50 | 1.69 ± 0.60 | 18.00 ± 2.70 |
| PVDF-TrFE/HAp 10% (P2) | 10.28 ± 2.49 | 1.71 ± 0.74 | 14.15 ± 5.28 |


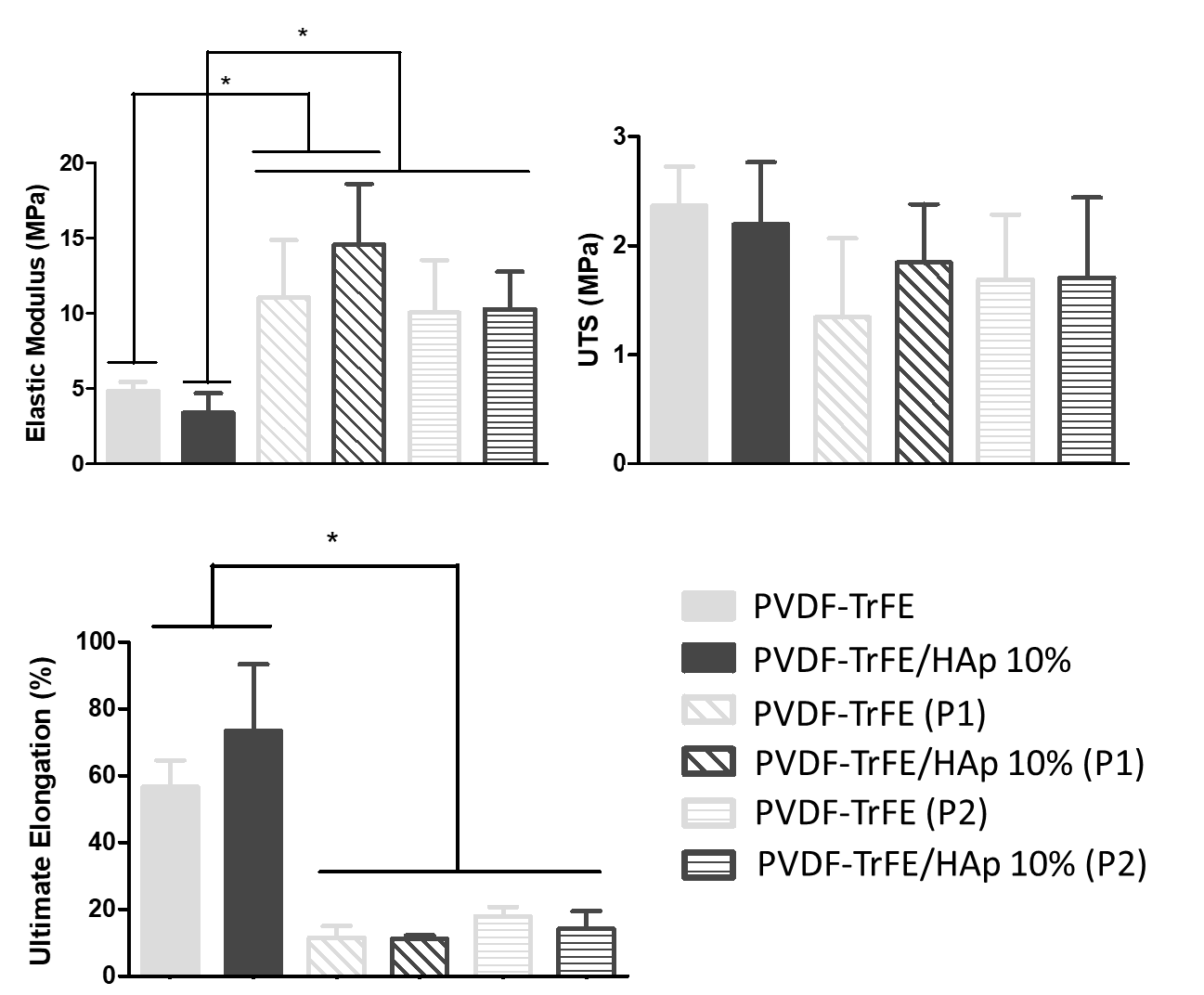


**Fig S7.** Mechanical properties of as-spun and annealed PVDF-TrFE and PVDF-TrFE/HAp 10% piezoelectric scaffolds obtained after tensile testing: elastic modulus (A), ultimate tensile strength (UTS) (B) and ultimate elongation (C). Five samples (n=5) were used in the analysis; *p < 0.05.


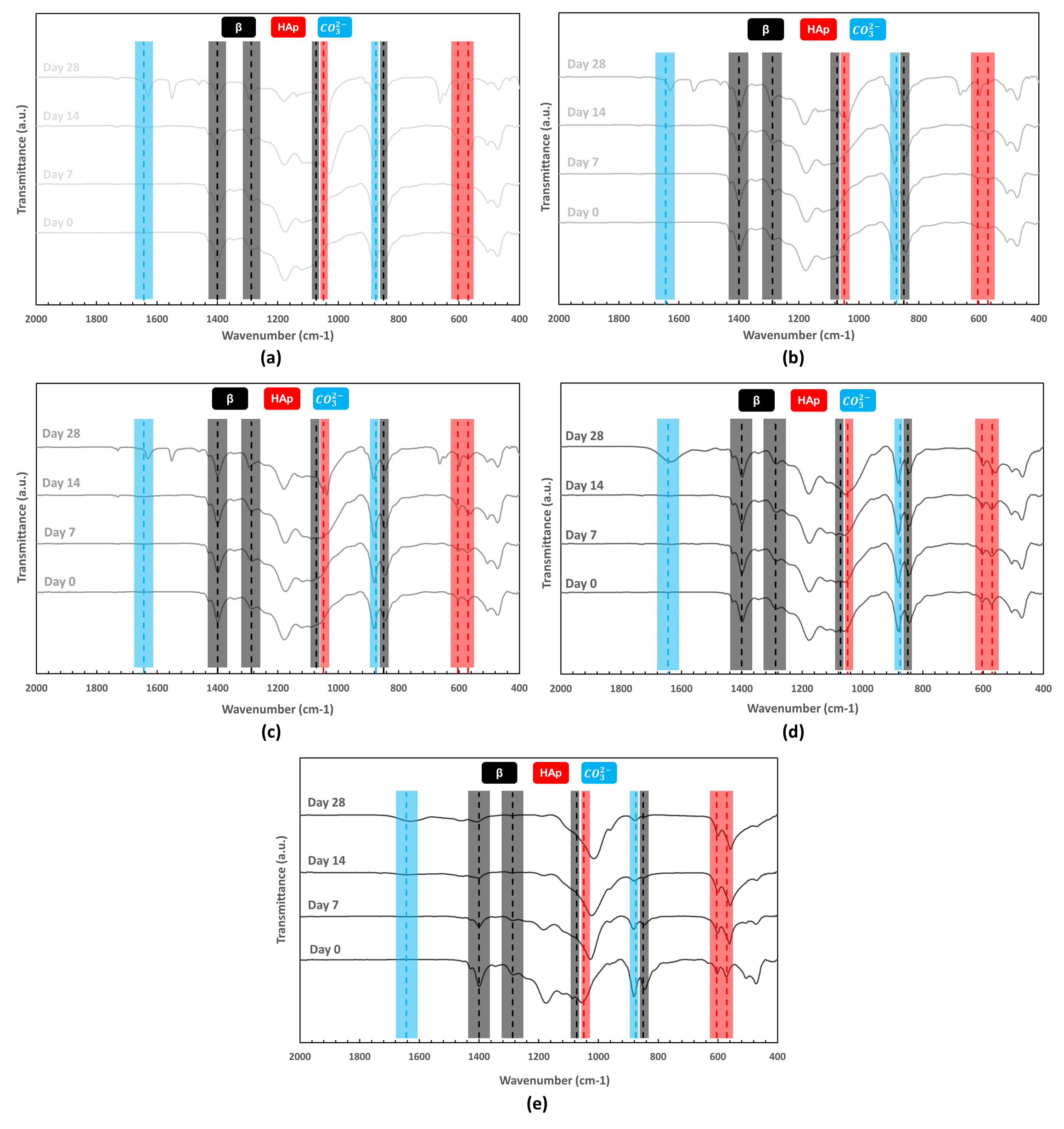
In Vitro Scaffold Mineralization

**Fig S8.** FTIR analysis of the PVDF-TrFE nanofibers – with 0% (a), 1% (b), 3% (c), 5% (d) and 10% (e) HAp – after 0, 7, 14 and 28 days of incubation in SBF.


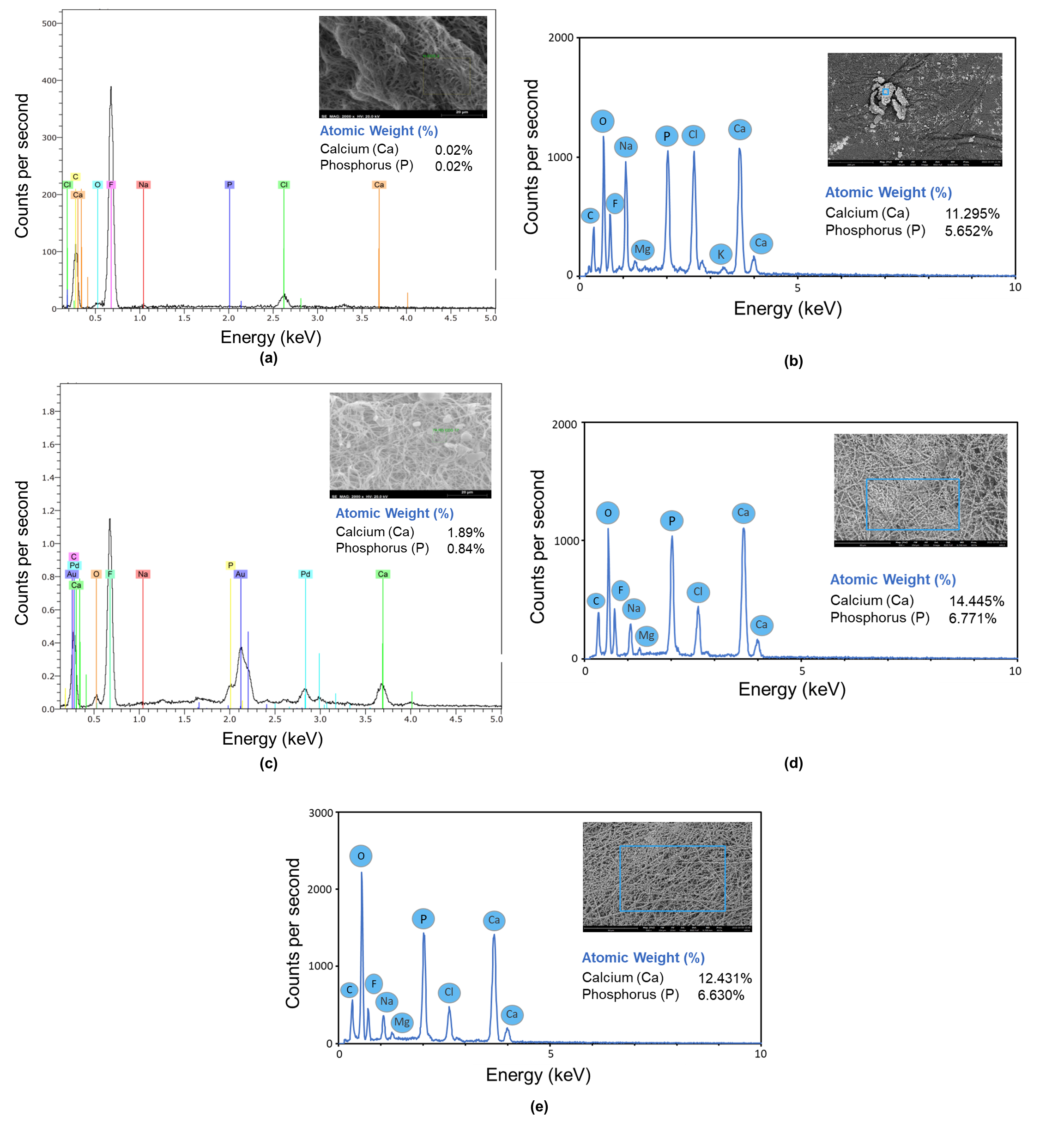
**Fig S9.** Elemental composition analysis (EDX) of the generated PVDF-TrFE/HAp nanofibers: PVDF-TrFE fibers without HAp (a) and PVDF-TrFE fibers with 1% (b), 3% (c), 5% (d) and 10% (e) HAp after 28 days incubation in SBF. SEM images of the spots where EDX analysis was conducted are presented inside the corresponding EDX spectrograms. The atomic percentage (Atomic%) of calcium (Ca) and phosphorus (P) present in each sample (used in Ca/P ratio computation) is highlighted.

In Vitro Cell Culture Studies

**Table S5.** Fold increase values in equivalent cell numbers measured at days 3, 7, 14 and 21 of cell culture in relation to the number of cells present on day 0.

|  | **Day 3** | **Day 7** | **Day 14** | **Day 21** |
| --- | --- | --- | --- | --- |
| PVDF-TrFE | 0.98 ± 0.10 | 1.07 ± 0.11 | 1.14 ± 0.08 | 2.03 ± 0.15 |
| PVDF-TrFE/HAp 1% | 1.09 ± 0.13 | 1.25 ± 0.16 | 1.43 ± 0.09 | 2.35 ± 0.10 |
| PVDF-TrFE/HAp 3% | 1.19 ± 0.17 | 1.23 ± 0.31 | 1.30 ± 0.03 | 2.40 ± 0.17 |
| PVDF-TrFE/HAp 5% | 1.06 ± 0.29 | 1.24 ± 0.22 | 1.41 ± 0.12 | 2.44 ± 0.15 |
| PVDF-TrFE/HAp 10% | 0.79 ± 0.19 | 1.08 ± 0.28 | 1.24 ± 0.16 | 2.24 ± 0.12 |

**
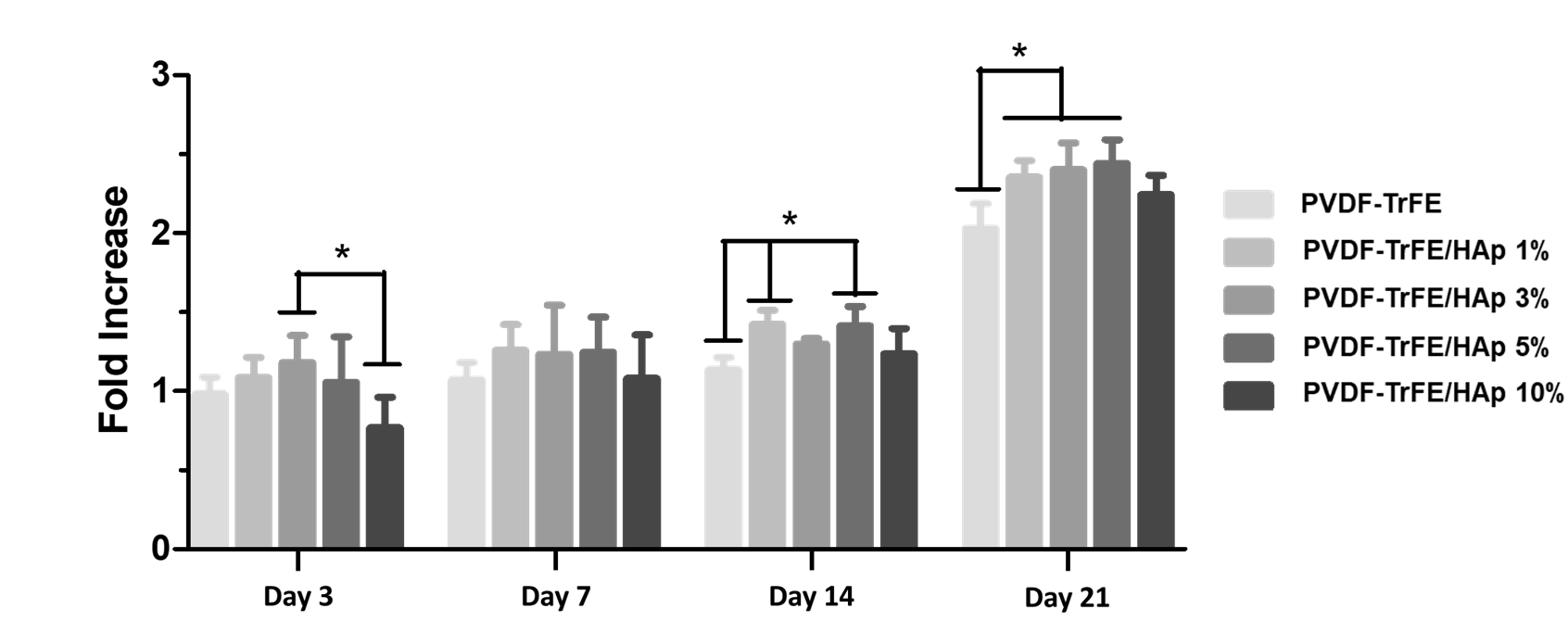
Fig S10.** Fold increase of equivalent cell numbers measured at days 3, 7, 14 and 21 of cell culture against the number of cells present on day 0; *p < 0.05.


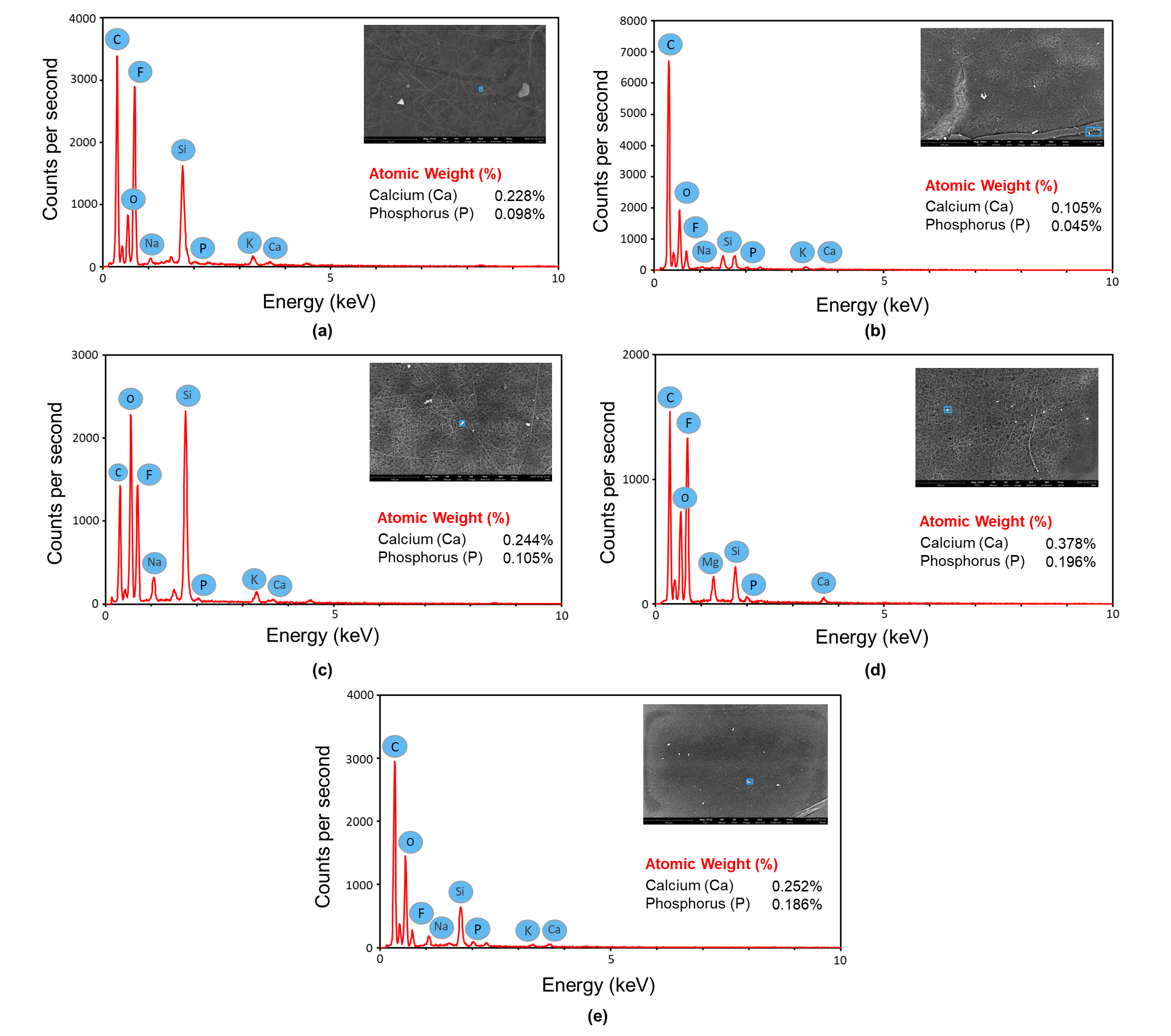
**Fig S11.** Elemental composition analysis (EDX) of the MSC-seeded PVDF-TrFE nanofibers (a) and PVDF-TrFE/HAp nanofibers with 1% (b), 3% (c), 5% (d) and 10% (e) HAp after 21 days of osteogenic differentiation. SEM images of the spots where EDX analysis was conducted are presented inside the respective EDX spectrograms. The atomic percentage (Atomic%) of calcium (Ca) and phosphorus (P) present in each sample (used in Ca/P ratio computation) is highlighted.
